# Performance Analysis and Design Principles of Wireless Mutual Broadcast Using Heterogeneous Transmit Power for Proximity-Aware Services

**DOI:** 10.3390/s24248045

**Published:** 2024-12-17

**Authors:** Taesoo Kwon, HyeonWoo Lee

**Affiliations:** 1 Department of Computer Science and Engineering, Seoul National University of Science and Technology (SeoulTech), Seoul 01811, Republic of Korea; 2 Department of Mobile Systems Engineering, Dankook University, Yongin 16890, Republic of Korea; woojaa@dankook.ac.kr

**Keywords:** proximity-based services, neighbor discovery, 6G IoT, heterogeneous devices, wireless broadcast, stochastic geometry, random access, power control

## Abstract

As proximity-aware services among devices such as sensors, IoT devices, and user equipment are expected to facilitate a wide range of new applications in the beyond 5G and 6G era, managing heterogeneous environments with diverse node capabilities becomes essential. This paper analytically models and characterizes the performance of heterogeneous random access-based wireless mutual broadcast (RA-WMB) with distinct transmit (Tx) power levels, leveraging a marked Poisson point process to account for nodes’ various Tx power. In particular, this study enables the performance of RA-WMB with heterogeneous Tx power to be represented in terms of the performance of RA-WMB with a common Tx power by deriving an equivalent Tx power based on the probability distribution of heterogeneous Tx power and the path loss exponent. This approach allows for an analytical and quantitative comparison of heterogeneous RA-WMB performance with the common Tx power configuration. Further, the study derives performance ratios among node groups with distinct Tx power levels and formulates an optimization problem to design a heterogeneous Tx power configuration that balances individual node group performance improvements with overall network performance, yielding the optimal Tx power configuration. A closed-form suboptimal transmission probability (TxPr) is also proposed to improve heterogeneous RA-WMB performance, providing an efficient alternative to iterative methods for the optimal TxPr. Numerical results demonstrate the accuracy of performance analysis and highlight the effectiveness of the proposed designs.

## 1. Introduction

With the advent of the 6th generation (6G) era and the increasing integration of artificial intelligence (AI) into everyday life, the demand for advanced and intelligent services continues to grow [1,2]. Among these, proximity-aware services stand out as a pivotal component, facilitating intelligent and context-sensitive interactions [1,3,4,5,6,7]. Proximity-aware services leverage spatial or geographical proximity to enable efficient and adaptive interactions among devices [3]. These services operate often without centralized coordination, relying on the capability of devices to detect, communicate with, and respond to nodes within a physical range [6,7,8,9]. The functionality typically initiates when devices broadcast their presence or identification information to nearby nodes, enabling detection and subsequent interaction. This process often involves the transmission of physical signaling patterns or broadcast messages (BMs) designed to reach a broad set of surrounding devices [10]. Such services are particularly advantageous in scenarios where localized interactions are critical, necessitating the exchange of information among proximate nodes. Their applications extend well beyond basic neighbor discovery [3], encompassing tasks such as geographic content sharing [4], real-time safety data dissemination in vehicle-to-vehicle (V2V) networks [5], and device-aware interactions enabled by Bluetooth Low Energy (BLE) [6,7]. They also support sensor-based environmental monitoring by enabling direct broadcast and data exchange among sensors [8], as well as unmanned aerial vehicle (UAV)-to-UAV communication [9] and context-aware interactions within social IoT systems [11]. Wireless mutual broadcast (WMB) [12,13,14] facilitates these applications by allowing devices to continuously advertise their presence, capabilities, or sensed data to nearby nodes while gathering similar information from surrounding devices. In AI-enhanced environments, WMB becomes particularly valuable by supporting real-time data exchange and facilitating localized intelligence essential for a wide array of proximity-based services, from autonomous vehicles to sensor-driven environmental monitoring and social IoT applications [1]. As 6G networks progress toward increasingly complex and AI-enabled structures, communication environments are evolving from homogeneous device arrangements to heterogeneous networks comprising diverse devices with various transmit (Tx) powers, priorities, and functional roles [1,2]. Deploying WMB in these multifaceted conditions introduces both challenges and opportunities for optimizing network performance and individual device experiences. In contrast to monotypic device environments, heterogeneous networks often exhibit variations in Tx power or communication priority, potentially impacting both overall network performance and creating differences in performance across diverse nodes. To address these diverse characteristics, this study analytically investigates network-wide performance and the disparities among nodes with distinct attributes in heterogeneous WMB networks, where nodes exhibit a range of Tx power capabilities and priority requirements.

In this context, the network-wide performance of WMB is collectively influenced by key factors such as node Tx power, radio propagation characteristics, spatial distribution of nodes, and varying service requirements [15,16]. Since the foundational work by [17,18] on wireless network performance, stochastic geometry has become essential for mathematically analyzing wireless networks and quantifying the effects of different operational parameters [19]. This method has been effectively applied across a wide array of network types, from multicell [20] and sensor networks [21] to vehicular [22] and satellite [23] systems, as well as emerging technologies in 6G, including massive MIMO and ultra-dense connectivity [19]. In WMB environments, stochastic geometry has supported research on random access protocol performance [12,13], transmission probability (TxPr) optimization [24], energy-efficient operation [25], and the integration of advanced methods such as full-duplex with self-interference cancellation [26], energy harvesting [27,28], and beamforming [29], as well as the analysis of correlated spatial node distributions [30]. Random access-based WMB (RA-WMB) protocols are particularly well suited for environments where nodes lack detailed knowledge of neighbors or need to broadcast messages (BMs) to unspecified nodes. Under the stochastic geometry framework, this study analytically examines network-wide performance in heterogeneous WMB networks employing random access and proposes design principles for optimizing TxPr and configuring Tx power.

### 1.1. Related Works

WMB involves tackling a range of technical challenges across its various applications [4,5,6,7,9,11,31,32,33,34,35,36], driven by high device densities, dynamic topologies, and energy constraints. Key issues include reducing interference from simultaneous broadcasts, improving energy efficiency in resource-limited networks, and achieving low latency for time-sensitive tasks such as emergency message dissemination. In heterogeneous environments, variations in energy budgets, duty cycles, and Tx power levels complicate operational efficiency and reliability [12,37,38]. To address these challenges, advanced analytical frameworks, such as stochastic geometry [12,13,14,25,26,27,28,29,30] and innovative techniques [4,5,6,7,9,11,12,31,32,33,34,35,36,37,38], including optimized transmission probability, energy-aware designs, and adaptive protocols, have been explored to enhance performance across different network conditions.

WMB has primarily been studied in the context of neighbor discovery, its most prominent application. In recent years, neighbor discovery technologies across various network environments, including IoT, V2V, and other emerging networks, have evolved to overcome unique technical obstacles inherent to each network, such as high device density, energy constraints, and dynamic topology changes. IoT environments face significant challenges in neighbor discovery due to the low capability of devices, imposing constraints on energy efficiency and processing power [6,7,31,32]. The high density of IoT devices exacerbates collisions, increasing latency and energy use. Advanced discovery techniques are needed to mitigate these issues while ensuring reliable and fast operation. The work of [6] surveyed BLE-based neighbor discovery, focusing on performance models and parameter optimizations to reduce collisions and improve energy efficiency. Optimizing advertising intervals, scanning windows, and synchronization settings was shown to effectively minimize latency, particularly in dense IoT networks. Probabilistic and simulation-based approaches were also highlighted as tools for fine-tuning BLE parameters in asynchronous environments. Ref. [7] introduced a periodic interval-based framework that decouples beacon transmission and scanning, unlike traditional slotted protocols. This decoupling enabled flexible scheduling, reducing collisions and discovery latency within fixed energy budgets. The method was particularly suited to dense, resource-constrained IoT deployments. In [31], reinforcement learning optimized neighbor discovery in the Internet of underwater things (IoUT) by dynamically adjusting beam scanning. The algorithm used previously discovered neighbors and handshake data to focus scans on likely directions, minimizing redundant operations. This approach improved energy efficiency and discovery latency in challenging underwater environments. In [32], a collaborative neighbor discovery method was proposed to address the challenges of energy efficiency and latency in IoT applications. The method coordinated sensors in a star topology by analytically optimizing duty cycles and wake-up schedules to minimize overlap in active periods and reduce redundant scanning. This approach ensured efficient operation under constrained energy budgets. Wireless networks composed of mobile nodes encounter significant challenges in neighbor discovery due to dynamic topology changes and the need to minimize latency and overhead. Neighbor discovery techniques have been adapted for UAV, vehicular, and mobile ad hoc networks to address these demands [9,33,34]. In [9], the challenges of dynamic topology and high mobility in UAV networks were addressed by a 3D neighbor discovery algorithm designed for directional antennas. The algorithm employed a skip scanning strategy with dynamic step size adjustment to optimize antenna rotation speed, mitigating latency caused by mobility and asynchronous operations. A Hilbert curve-based scanning path further reduced mechanical rotation overhead, ensuring efficient spatial coverage and reliable discovery. The work of [33] proposed the neighbor discovery algorithm combining a gossip mechanism, multipacket reception (MPR), and roadside unit (RSU)-assisted sensing to address high mobility and dynamic topology in vehicular networks. The gossip mechanism propagated both direct and indirect neighbor information during handshakes, ensuring rapid updates. MPR reduced collisions by enabling simultaneous processing of overlapping packets, while RSU sensing focused scanning on nonempty beams to improve convergence speed. In [34], a beam configuration algorithm for neighbor discovery in mobile ad hoc networks with directional antennas was introduced to address challenges like dynamic topology and mobility. The algorithm combined personalized federated learning and deep reinforcement learning to dynamically optimize beam configurations based on local and aggregated network data, effectively minimizing discovery latency and computational overhead.

WMB is not limited to neighbor discovery but has become essential for a wide range of services, including local data broadcasting and message dissemination. Despite its versatility, WMB-based systems must address critical challenges, such as reducing redundancy and improving delivery success in networks with variable node density and mobility. Moreover, minimizing latency is essential for all broadcast scenarios, especially for emergency messages, where immediate delivery is paramount due to their urgency. For example, several studies explored how WMB could be adapted to meet these challenges across different applications [4,5,11,35,36]. In mobile IoT networks, ref. [4] proposed a neighbor-based probabilistic broadcast protocol to reduce redundancy and improve delivery success in environments with high mobility and variable node density. The protocol calculated rebroadcast delays based on the number of uncovered neighbors and adjusted rebroadcast probabilities. This ensured that transmissions prioritized nodes likely to reach more uncovered neighbors. In cellular vehicular-to-everything networks, ref. [5] proposed a protocol to tackle challenges such as high mobility, packet loss, and broadcast storms in dense traffic scenarios. The method selected optimal forwarders for emergency message dissemination by evaluating metrics such as forwarding probability, transmission time, and vehicle speed, using a crow search algorithm enhanced with chaotic theory. Ref. [11] surveyed methods addressing data dissemination challenges in mobile social networks, focusing on their dynamic topologies and intermittent connectivity, where frequent changes in node positions and the formation of isolated clusters disrupted consistent communication. To overcome these issues, the survey highlighted strategies leveraging social properties such as tie strength, centrality, and community affiliation. These strategies predicted optimal forwarding paths by analyzing social behaviors, enabling nodes to prioritize relays likely to maximize delivery success. In massive IoT networks, ref. [35] proposed a location-aware forwarding scheme to minimize redundant transmissions and improve energy efficiency in large-scale data dissemination, particularly for emergency broadcasts. The scheme hierarchically organized devices by geographic location and assigned forwarding roles to nodes strategically positioned to maximize road coverage, reducing unnecessary broadcasts and conserving energy. In vehicular ad hoc networks, ref. [36] proposed dissemination protocols to reduce message collisions and enhance delivery reliability in highly dynamic and mobile environments. The approach combined distance-based selection with road geometry to identify relay nodes strategically positioned to maximize coverage and minimize redundant retransmissions. By aligning message forwarding with road layouts and vehicle mobility patterns, these protocols improved communication efficiency and reduced collisions.

Research on WMB in environments where heterogeneous nodes coexist remains relatively scarce, with the few existing studies primarily focused on overcoming significant challenges stemming from the coexistence of nodes with different energy budgets [37,38], duty cycles, or Tx power levels [12]. These disparities introduce inefficiencies, increase latency, and reduce reliability in discovery and broadcasting operations. The work of [37] addressed the issue of energy heterogeneity, where nodes with varying battery capacities struggle to align their discovery or broadcast intervals. The study proposed a duty–cycle adjustment technique that synchronizes discovery intervals with the energy capacities of individual nodes. This solution reduced unnecessary wake-ups, improved energy efficiency, and extended the operational life of heterogeneous sensor networks. Another study, ref. [38], tackled the problem of accommodating heterogeneous IoT devices with asymmetric energy budgets. The proposed solution employed an asymmetric discovery model that divided discovery slots into pure sending and pure listening periods, dynamically adjusted based on nodes’ energy availability. This model significantly reduced discovery latency while improving overall energy efficiency. In contrast, ref. [12] focused on the heterogeneous WMB scenarios, where node groups coexist with different Tx power levels. This study specifically analyzed how retransmission-based error control mechanisms, such as Chase combining and incremental redundancy, affected the performance and reliability of local broadcasts in these power-diverse configurations.

Stochastic geometry has been widely applied to analyze and optimize WMB systems, focusing on key challenges such as mitigating interference caused by simultaneous BM transmissions, improving energy efficiency, and addressing security concerns in spatially distributed networks. This quantitative framework provides tools for assessing the impact of node spatial distribution and designing parameters like TxPr and Tx power to enhance performance under diverse network conditions. In [13], stochastic geometry was applied to analyze the performance of neighbor discovery, equivalent to WMB in this paper, in networks where nodes were independently and randomly distributed, modeled using a homogeneous Poisson point process (HPPP). This study examined random access wireless networks operating under random distribution, offering analytical designs to optimize TxPr and data rate for BM to maximize discovery performance. In [13], a suboptimal TxPr applicable to general path loss exponents (PLEs) was proposed, along with a mathematical analysis of the properties of the optimal TxPr, but this suboptimal solution was expressed as the root of an equation rather than in closed form. Expanding on RA-WMB, subsequent studies have focused on power-efficient designs. Ref. [25] investigated the joint design of TxPr and Tx power to minimize network-wide power consumption. Additional works, such as [27,28], introduced energy harvesting within RA-WMB, designing TxPr that accounts for simultaneous transmitting, receiving, and harvesting actions. Further, ref. [29] explored the interactions between the number of antennas, Tx power, and TxPr in RA-WMB networks with directional beaming capabilities, aiming to minimize energy use. Beyond power consumption, security and correlated spatial node distribution in RA-WMB have also been addressed. Studies have expanded to consider RA-WMB network configurations with passive eavesdroppers analyzing secrecy performance and TxPr design under eavesdropping threats [14], as well as RA-WMB networks with spatial clustering of nodes to assess network-wide spatial performance [30]. Studies on heterogeneous RA-WMB networks based on stochastic geometry have considered scenarios where nodes exhibit different transceiving characteristics. Ref. [12] examined a local broadcasting network, akin to RA-WMB, where node groups with different Tx power levels coexisted. This study quantified the number of neighbor nodes from which BMs could be successfully received in an interference-limited configuration and offered insights into error control and Tx power configurations for cognitive radio environments. And, ref. [26] analyzed the spatial performance of heterogeneous RA-WMB networks with coexisting half-duplex and full-duplex nodes, deriving optimal TxPr through a mathematical analysis of these heterogeneous configurations.

To the best of our knowledge, although various studies have been proposed on different aspects of RA-WMB, no research has analytically and quantitatively investigated the beneficial and adverse impacts of distinct Tx power in RA-WMB networks on netwide performance compared with RA-WMB networks with common Tx power, nor focused on optimizing performance in this context.

### 1.2. Contributions and Organization

This paper analytically studies the network-wide performance of a heterogeneous RA-WMB, consisting of nodes that operate with distinct Tx power levels under a common TxPr. The investigation addresses both the overall network perspective and the performance characteristics of individual node groups with distinct Tx power levels. While more sophisticated access methods, such as carrier sense multiple access (CSMA), could further enhance overall performance, this study employs slotted Aloha as the access scheme because it provides a straightforward baseline for various random access methods and facilitates clearer insights into network performances and design strategies. Although the combined application of distinct Tx power levels and distinct TxPr configurations could enable finer performance differentiation among node groups, this study focuses solely on heterogeneous Tx power scenarios with a common TxPr across all nodes for analytical simplicity. Notably, the study demonstrates that even with a common TxPr, performance prioritization among nodes can be effectively achieved through distinct Tx power configurations alone. The investigation of advanced access methods and joint designs for heterogeneous TxPr-Tx power configurations is reserved for future work. Within this analytical framework, the key contributions of this paper are highlighted below.

1. *Elegant performance expression of RA-WMB with heterogeneous Tx power*: This study analyzes the network-wide performance of RA-WMB with heterogeneous Tx power configurations by applying a marked HPPP, treating the Tx power of each node as a mark. By using the equivalent Tx power, derived from the probability distribution of heterogeneous Tx powers and the PLE, the performance of the heterogeneous RA-WMB network is represented as that of an RA-WMB network with a common Tx power, where all nodes transmit at this equivalent Tx power. This simplified representation enables a more straightforward analysis of heterogeneous RA-WMB performance by leveraging valuable insights from conventional studies on RA-WMB with common Tx power.
2. *Analytical quantification of beneficial and adverse impacts of heterogeneous Tx power*: This study analytically quantifies the impact of heterogeneous Tx power on RA-WMB performance. By comparing the maximum achievable performance of RA-WMB with heterogeneous Tx power to that of RA-WMB with a common Tx power, each at its optimal TxPr, it demonstrates that heterogeneous Tx power offers no improvement in overall network performance. Performance loss due to heterogeneous Tx power is greatest in coverage-limited scenarios and decreases with increasing interference, approaching the performance of common Tx power RA-WMB in interference-limited scenarios. Further, the study explicitly derives the performance ratios among heterogeneous nodes with different Tx power levels to quantify the performance disparity among nodes.
3. *Design and optimization of Tx power configuration problem for prioritizing node groups*: This study proposes a heterogeneous Tx power configuration strategy to boost the performance of specific node groups within an acceptable range of overall performance loss due to heterogeneous Tx power. To achieve this, an optimization problem is formulated to derive the optimal Tx power configuration. This optimal Tx power is obtained through bisection search, utilizing the optimal value of common TxPr at each iteration to achieve the best possible performance. To simplify the process, the iterative calculation of the optimal TxPr is replaced by a closed-form suboptimal TxPr proposed in this study. This substitution performs effectively with minimal performance degradation under practical operating conditions.

This study differs significantly from [12,24]. Ref. [12] analyzed RA-WMB performance in environments with nodes of different Tx powers but focused only on interference-limited scenarios, deriving performance based on the signal-to-interference ratio (SIR). The proposed Tx power allocation prioritized maintaining the primary group’s performance at a fixed level, with its Tx power pre-determined, while the secondary group’s Tx power was adjusted accordingly, without jointly optimizing the Tx powers of both groups. In contrast, this study extends the analysis to include low and moderate node density scenarios alongside high-density environments by evaluating performance based on the signal-to-interference-plus-noise ratio (SINR). It analytically quantifies the overall system performance impact of Tx power disparity and specifically designs the Tx powers for two coexisting node groups through joint optimization. A closed-form common TxPr is derived for an arbitrary number of node groups to maximize overall performance while simultaneously ensuring the maximization of individual group performance. On the other hand, ref. [24] derived a suboptimal TxPr aimed at improving RA-WMB performance under SINR-based analysis. However, it did not provide a closed-form solution and was limited to common Tx power configurations, excluding consideration of heterogeneous node environments with distinct Tx powers. The notations in this paper are described in Table 1, which also lists the parameter values used for the numerical results in Section 5.

The remainder of this paper is organized as follows: Section 2 describes the heterogeneous RA-WMB models and defines the network-wide performance metrics, including both the overall performance and the performance of individual node groups with distinct Tx powers. Section 3 analytically characterizes the quantitative impact of heterogeneous Tx power configurations, focusing on overall RA-WMB performance, performance disparities among individual node groups with distinct Tx power levels, and the overall performance loss caused by these heterogeneous power levels. Section 4 formulates and solves an optimization problem for Tx power configurations to differentiate the performance of specific node groups while also deriving a closed-form common TxPr to enhance both overall and individual group performance. Section 5 validates and discusses the proposed results through numerical simulations. Finally, Section 6 concludes the paper.

*Notations*: $E_{X}\left[f\left(X\right)\right]$ denotes the expected value of f(X) with respect to *X*. P[E] represents the probability of the event *E*. 1[Y=y] is the indicator function, which equals 1 if Y=y and 0 otherwise. [K] denotes the set {1,2,⋯,K}. $R^{d}$ represents the *d*-dimensional real number space.

## 2. Performance Models for Heterogeneous RA-WMB with Diverse Transmit Power

This section describes heterogeneous RA-WMB models, where nodes operate at distinct Tx power levels, and defines the metrics for evaluating network-wide performance.

### 2.1. System Models

In RA-WMB networks with heterogeneous Tx power configurations across nodes, nodes are assumed to be spatially distributed according to an HPPP, denoted as Φ with density λ. This assumption provides a mathematically tractable model where nodes are distributed randomly and independently over the plane, ensuring that the presence of one node does not influence the locations of others. The node density denoted by λ represents the average number of nodes per unit area. The combination of HPPP node distribution with slotted Aloha-based random access further simplifies analysis. This is because transmitting nodes can be modeled as an independently thinned HPPP, a property guaranteed by the displacement theorem [15], which states that independent thinning of an HPPP results in another HPPP. While more sophisticated access mechanisms like CSMA increase inter-node spacing and reduce interference, in WMB, where all broadcasting nodes transmit meaningful data, it also diminishes the received signal power from desired transmitting nodes, leading to varying performance depending on environmental factors. For instance, ref. [39] analyzed RA-WMB under repulsive node distributions and found that while environmental factors may slightly alter performance, the optimal transmitting node density for maximizing WMB performance remains similar to that of an HPPP. This suggests that even with CSMA, the overall performance trends may not deviate significantly. It is also important to consider non-uniform node distributions, such as clustered patterns, which can occur in practical deployments. Clustered distributions, like those modeled by a Thomas point process, may increase interference due to closer node proximity but can simultaneously improve desired signal reception in WMB. For example, ref. [30] analyzed RA-WMB performance under a clustered node distribution modeled by a Thomas point process. Although the analysis was conducted in a zero-noise environment and reported results were limited to specific scenarios and parameters, the performance trends were inferred to closely align with those observed for HPPP. This suggests that the HPPP-based model, despite its simplifying assumptions, provides a meaningful baseline for analyzing WMB performance. As a result, HPPP-based analysis offers a robust foundation for understanding WMB systems while enabling insights into baseline performance. Future research can expand on this by exploring more advanced access mechanisms and diverse node distribution models to better capture the complexities of real-world scenarios.

**Figure 1:**
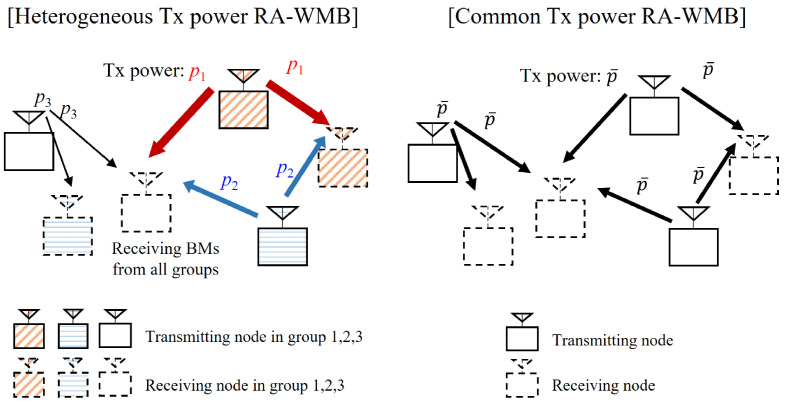
Comparison of RA-WMB with heterogeneous and common Tx power configurations.

In this HPPP model, both node *i* and its location are denoted by $X_{i}$, with a slight abuse of notation. Further, this study considers a scenario where Tx power values are independent across nodes, with each node operating at a fixed Tx power level. Figure 1 provides a comparative illustration of RA-WMB scenarios with heterogeneous and common Tx power configurations. To analyze scenarios involving node-specific characteristics, a marked HPPP is employed. In a marked HPPP, each node $X_{i}$ in the base point process Φ is associated with an additional random variable, or mark, $m_{i}$, representing a node-specific property, i.e., its Tx power. The marked PPP is expressed as $\hat{\Phi }≜\left(X_{i},m_{i}\right)$, where the spatial distribution of nodes follows an HPPP, and the marks $m_{i}$ are modeled as independent and identically distributed (iid) random variables that are independent of Φ. This independence ensures that the spatial distribution and node-specific attributes remain statistically decoupled, retaining the analytical tractability of the base HPPP. The set of possible marks, M, may represent either a continuous range of values or a discrete set, without any restriction. This flexibility makes the marked HPPP particularly suitable for studying the WMB systems where heterogeneity among nodes plays a critical role in performance evaluation. Therefore, $m_{i}$ is an i.i.d. random variable satisfying $m_{i}\geq 0$ and $m_{i}\in M$, where M denotes the set of possible marks.

Meanwhile, each node transmits its BM with TxPr ν, using a resource block (RB) randomly selected from *K* orthogonal RBs. With probability 1−ν, a node attempts to receive BMs from other nodes across all *K* RBs. While controlling both TxPr and Tx power levels across nodes could enhance performance differentiation, this study focuses on distinct Tx power levels alone to effectively distinguish performance among nodes. A common TxPr, denoted as ν, is thus applied uniformly across all nodes, leaving the potential differentiation of TxPr values among nodes for future work.

The model assumes wireless channels where transmitted signals undergo standard power loss with a PLE α and Rayleigh fading with a unit mean. Although the Nakagami-*m* fading model could capture a broader range of short-term fading scenarios, it does not significantly impact network-wide performance trends or design principles [13]; hence, Rayleigh fading is adopted here for analytical convenience. Furthermore, when nodes are distributed spatially according to an HPPP, the influence of iid shadowing can be modeled as an equivalent HPPP [40]. Therefore, the findings in this paper can be readily extended to account for shadowing effects, consistent with the approaches in [13,24], even though shadowing is not explicitly analyzed here.

The transceiving status of node *i* is defined as $T_{i}$, assigned a value of 1 if $X_{i}$ transmits its BM and 0 if it receives BMs from other nodes. And the interfering status of node *j* with respect to node *i*, denoted by $I_{i,j}$, is set to 1 if $X_{j}$ transmits on the same RB as $X_{i}$ and 0 otherwise. The SINR at the typical node $X_{o}$, located at the origin, when attempting to decode the BM from $X_{i}$ with Tx power $m_{i}$, is given by

(1) $$\begin{array}{c} \begin{array}{c} \Xi \left(X_{i},m_{i}\right)=\frac{m_{i}h_{i}{\left|X_{i}\right|}^{-\alpha }}{I\left(X_{i}\right)+\sigma^{2}}, \end{array} \end{array}$$

where $I\left(X_{i}\right)≜\sum_{j:\left(X_{j},m_{j}\right)\in \hat{\Phi }\setminus \left\{\left(X_{o},m_{o}\right),\left(X_{i},m_{i}\right)\right\}}1\left[I_{i,j}=1\right]m_{j}h_{j}{\left|X_{j}\right|}^{-\alpha }$ represents the interference term. Here, $\sigma^{2}≜\frac{{\tilde{\sigma }}^{2}}{A}$ with ${\tilde{\sigma }}^{2}$ and *A*, representing the thermal noise power and path loss gain at unit distance, respectively. And $h_{i}$ denotes the Rayleigh fading gain from $X_{i}$ to $X_{o}$, and $|X_{j}|$ denotes the distance of $X_{j}$ from the origin. In this study, successful BM decoding is achieved when the received SINR exceeds a threshold value, ξ.

### 2.2. Network-Wide Heterogeneous RA-WMB Performances

This paper defines and evaluates the network-wide performance of heterogeneous RA-WMB from two perspectives: the overall performance encompassing all nodes and the performance of node groups operating at specific Tx power levels. To clearly describe the heterogeneous Tx power configuration for evaluating these performance metrics, Ω is defined as follows:

(2) $$\begin{array}{c} \begin{array}{c} \Omega ≜\{\left(p_{g},\rho_{g}\right):\rho_{g}=P\left[m_{i}=p_{g}\right]\;\mathrm{for}\;\left(X_{i},m_{i}\right)\in \hat{\Phi },p_{g}\in M,\;\mathrm{and}\;g\in \left[G\right]\}, \end{array} \end{array}$$

where M represents the set of possible Tx power levels, as defined in Section 2.1. When considering *G* possible Tx power levels, M can be expressed as $\left\{p_{1},p_{2},\cdots ,p_{G}\right\}$. Here, *G* does not necessarily need to be finite. Thus, the heterogeneous Tx power configuration of nodes in the RA-WMB network can be represented through $\hat{\Phi }$ and Ω.

To begin with, from the perspective of overall performance, the average number of BMs successfully received by the typical node across all *K* RBs, denoted by S(Ω,ν), is defined for a given Ω and ν as follows:

(3) $$\begin{array}{c} \begin{array}{c} S\left(\Omega ,\nu \right)≜E\left[1\left[T_{0}=0\right]\;\sum_{i:\left(X_{i},m_{i}\right)\in \hat{\Phi }\setminus \left\{\left(X_{o},m_{o}\right)\right\}}\;1\left[T_{i}=1,\Xi \left(X_{i},m_{i}\right)>\xi \right]\right]. \end{array} \end{array}$$

On the other hand, the performance of node groups operating at specific Tx power levels is measured by counting the BMs successfully received by the typical node from nodes with a specific transmit power value $m_{i}=p_{g}$. This performance for a given Ω and ν is defined as follows:

(4) $$\begin{array}{c} \begin{array}{c} S_{g}\left(\Omega ,\nu \right)≜E\left[1\left[T_{0}=0\right]\;\sum_{i:\left(X_{i},m_{i}\right)\in \hat{\Phi }\setminus \left\{\left(X_{o},m_{o}\right)\right\}}\;1\left[T_{i}=1,m_{i}=p_{g},\Xi \left(X_{i},m_{i}\right)>\xi \right]\right]. \end{array} \end{array}$$

For instance, if $M=\{p_{1},p_{2},\cdots ,p_{G}\}$, then S and $S_{g}$ naturally have the following relationship:

(5) $$\begin{array}{c} \begin{array}{c} S=\sum_{g=1}^{G}S_{g}. \end{array} \end{array}$$

In this study, the Tx power of nodes in $\hat{\Phi }$ is determined independently and identically according to the heterogeneous Tx power configuration Ω. This allows the Tx power of these nodes to be collectively represented by random variable *M*. Consequently, the network-wide average Tx power in this heterogeneous RA-WMB network can be expressed as

(6) $$\begin{array}{c} \begin{array}{c} \bar{p}≜E_{M}\left[M\right]. \end{array} \end{array}$$

To evaluate the performance of S under a heterogeneous Tx power configuration relative to a scenario where all nodes transmit at a common power level, the performance when all nodes operate at common Tx power $\bar{p}$ is defined as $S_{E}$, like

(7) $$\begin{array}{c} \begin{array}{c} S_{E}\left(\bar{p},\nu \right)≜S\left(\left\{\left(\bar{p},1\right)\right\},\nu \right). \end{array} \end{array}$$

Using performance metrics such as S and $S_{E}$, this study analytically examines the impact of heterogeneous Tx power on RA-WMB performance compared with common Tx power. Further, it explores methods to support a high-priority node group through differentiated Tx power levels.

## 3. Spatial Performance Analysis of Heterogeneous RA-WMB Network

This section analytically characterizes the performance of heterogeneous RA-WMB networks and examines the impact of distinct Tx power configurations on network performance.

### 3.1. Overall Performance for Heterogeneous RA-WMB Networks

The following results address S in (3), representing the average number of BMs successfully received by a typical node from all other nodes.

**Theorem** **1.** *Given a heterogeneous Tx power configuration* **Ω** *and TxPr ν, S in* (3) *is expressed as*

(8) $$\begin{array}{c} \begin{array}{cc} \; & S\left(\Omega ,\nu \right)=\pi \lambda \nu \left(1-\nu \right)E_{M}\left[M^{2/\alpha }\right]\int_{0}^{\infty }e^{-\xi \sigma^{2}u^{\alpha /2}-\pi \frac{\lambda \xi^{2/\alpha }}{K\Delta (\alpha )}E_{M}\left[M^{2/\alpha }\right]\nu u}du, \end{array} \end{array}$$

*where $\Delta \left(\alpha \right)≜\frac{\sin(2\pi /\alpha )}{2\pi /\alpha }$. This S is equivalent to the performance in a scenario where all nodes operate at a common Tx power of $E_{M}{\left[M^{2/\alpha }\right]}^{\alpha /2}$, i.e.,*

(9) $$\begin{array}{c} \begin{array}{cc} \; & S\left(\Omega ,\nu \right)=S_{E}\left({\bar{p}}_{E},\nu \right)\;where\;\;\;\;\;{\bar{p}}_{E}≜E_{M}{\left[M^{2/\alpha }\right]}^{\alpha /2}. \end{array} \end{array}$$

*Further, S is lower bounded as follows:*

(10) $$\begin{array}{c} \begin{array}{c} S\left(\Omega ,\nu \right)>\tilde{S}\left(\Omega ,\nu \right), \end{array} \end{array}$$

(11) $$\begin{array}{c} \begin{array}{c} where\;\tilde{S}\left(\Omega ,\nu \right)≜\frac{\pi \lambda }{{\left(\xi \sigma^{2}\right)}^{2/\alpha }}\Gamma \left(1+\frac{2}{\alpha }\right)E_{M}\left[M^{2/\alpha }\right]\nu \left(1-\nu \right)e^{-\frac{\pi \lambda }{K{\left(\sigma^{2}\right)}^{2/\alpha }\tilde{\Delta }\left(\alpha \right)}E_{M}\left[M^{2/\alpha }\right]\nu }, \end{array} \end{array}$$

*and $\tilde{\Delta }\left(\alpha \right)≜\Delta \left(\alpha \right)\frac{\Gamma (2/\alpha )}{\Gamma (4/\alpha )}=\frac{\sin(2\pi /\alpha )}{2\pi /\alpha }\frac{\Gamma (2/\alpha )}{\Gamma (4/\alpha )}$.*

**Proof.**  See Appendix A. □

In Theorem 1, the expression in (9) provides a key result by equating the performance of heterogeneous Tx power configurations to that of an equivalent common Tx power, $E_{M}{\left[M^{2/\alpha }\right]}^{\alpha /2}$. This equivalence holds in terms of overall performance, effectively treating the system as if all nodes transmit at the equivalent Tx power. Such a perspective allows for the direct application of existing analytical results for common Tx power configurations, as demonstrated in [13,14,24]. For example, [14,24] provided closed-form lower bounds for RA-WMB performance, which can be readily adapted for analyzing heterogeneous Tx power systems. Here, ref. [14] studied physical layer security performance for RA-WMB; however, by setting the eavesdropper density to zero, its results become applicable to RA-WMB performance analysis under a common Tx power configuration. Although these studies proposed suboptimal TxPr designs to maximize RA-WMB performance based on these lower bounds, they did not derive a closed-form expression for suboptimal TxPr. To address this gap, this study derives a new lower bound, as defined in (11), which is utilized in Section 4.1 to derive a closed-form suboptimal TxPr.

Further, the equivalence in (9) provides a foundational framework for subsequent sections. It facilitates the analysis of the performance of node groups with distinct Tx power levels in Section 3.2, the evaluation of potential performance gains over the common Tx power case in Section 3.3, and the derivation of key results for designing heterogeneous Tx power configurations in Section 4.2.

Although S(Ω,ν) in (8) still requires an integral expression, closed-form expressions can be derived for the two extreme scenarios of interference-limited and coverage-limited cases, similar to the results in [13,24].

**Corollary** **1.** *In an interference limited scenario with $\sigma^{2}\to 0$,*

(12) $$\begin{array}{c} \begin{array}{cc} \; & \lim_{\sigma^{2}\to 0}S\left(\Omega ,\nu \right)=\frac{K\Delta (\alpha )}{\xi^{2/\alpha }}\left(1-\nu \right)<\frac{K\Delta (\alpha )}{\xi^{2/\alpha }}, \end{array} \end{array}$$

*which indicates that $\lim_{\sigma^{2}\to 0}S$ approaches its upper bound, given by $\frac{K\Delta (\alpha )}{\xi^{2/\alpha }}$, as ν goes to 0.*

*In contrast, in a coverage-limited scenario with no interference, which can be modeled by K→∞,*

(13) $$\begin{array}{c} \begin{array}{cc} \lim_{K\to \infty }S\left(\Omega ,\nu \right)\; & =\frac{\pi \lambda }{{\left(\xi \sigma^{2}\right)}^{2/\alpha }}\Gamma \left(1+\frac{2}{\alpha }\right)E_{M}\left[M^{2/\alpha }\right]\nu \left(1-\nu \right)\\ \; & \leq \frac{\pi \lambda }{4{\left(\xi \sigma^{2}\right)}^{2/\alpha }}\Gamma \left(1+\frac{2}{\alpha }\right)E_{M}\left[M^{2/\alpha }\right], \end{array} \end{array}$$

*which implies that the maximum of $\lim_{K\to \infty }S$ is $\frac{\pi \lambda }{4{\left(\xi \sigma^{2}\right)}^{\alpha /2}}\Gamma \left(1+\frac{2}{\alpha }\right)E_{M}\left[M^{2/\alpha }\right]$, achieved at $\nu =\frac{1}{2}$.*

**Proof.**  When ${\bar{p}}_{E}≜E_{M}{\left[M^{2/\alpha }\right]}^{\alpha /2}$, from (9), it holds that $\lim_{\sigma^{2}\to 0}S\left(\Omega ,\nu \right)=\lim_{\sigma^{2}\to 0}S_{E}\left({\bar{p}}_{E},\nu \right)$ and $\lim_{K\to \infty }S\left(\Omega ,\nu \right)=\lim_{K\to \infty }S_{E}\left({\bar{p}}_{E},\nu \right)$.

Therefore, by using the equivalent common Tx power of ${\bar{p}}_{E}$, the closed-form expressions, upper bounds, and optimal TxPr for $\lim_{\sigma^{2}\to 0}S\left(\Omega ,\nu \right)$ and $\lim_{K\to \infty }S\left(\Omega ,\nu \right)$ in (12) and (13) can be readily derived from Equations (3) and (4) in [24]. □

### 3.2. Performance Differentiation Through Heterogeneous Transmit Power Levels

This subsection quantitatively examines the performance differences among nodes with distinct Tx power levels. For a clearer comparison, a finite set of *G* Tx power levels, $M=\{p_{1},p_{2},\cdots ,p_{G}\}$, is considered, where the spatial density of nodes with each Tx power level $p_{g}$ is represented by $\lambda_{g}$, with g∈[G]. According to [15] (Equation (7.5)), $P[m_{i}=p_{g}]$ for stationary marked HPPP $\hat{\Phi }$ can be expressed as follows:

(14) $$\begin{array}{c} \begin{array}{c} \rho_{g}=P\left[m_{i}=p_{g}\right]=\frac{\lambda_{g}}{\lambda }\;\mathrm{for}\;g\in [G]. \end{array} \end{array}$$

In this heterogeneous Tx power configuration environment, the following result analytically expresses $S_{g}$ in (5).

**Corollary** **2.** *When $\Omega =\Omega_{G}≜\left\{\left(p_{1},\rho_{1}\right),\left(p_{2},\rho_{2}\right),\cdots \left(p_{G},\rho_{G}\right)\right\}$, where $\rho_{g}=\frac{\lambda_{g}}{\lambda }$ for g∈[G],*

(15) $$\begin{array}{c} \begin{array}{c} S_{g}\left(\Omega_{G},\nu \right)=\frac{\rho_{g}p_{g}^{2/\alpha }}{E_{M}\left[M^{2/\alpha }\right]}S\left(\Omega_{G},\nu \right)\;=\frac{\rho_{g}p_{g}^{2/\alpha }}{E_{M}\left[M^{2/\alpha }\right]}S_{E}\left({\bar{p}}_{E},\nu \right), \end{array} \end{array}$$

*where ${\bar{p}}_{E}=E_{M}{\left[M^{2/\alpha }\right]}^{\alpha /2}={\left(\sum_{g=1}^{G}\rho_{g}p_{g}^{2/\alpha }\right)}^{\alpha /2}$.*

*The result of* (15) *implies that the ratio of $S_{p_{g}}$ values corresponds to the ratio of $\rho_{g}p_{g}^{2/\alpha }$ values.*

**Proof.**  See Appendix B. □

The results of Corollary 2 demonstrate that the performance ratio between node groups with distinct Tx powers is independent of ν and is simply determined by the ratio of $\rho_{g}p_{g}^{2/\alpha }$ values. Moreover, the overall performance is succinctly represented as the performance of RA-WMB with an equivalent common Tx power, ${\bar{p}}_{E}$. This representation decouples the evaluation of overall performance loss from the analysis of performance disparity among node groups; i.e., the equivalent Tx power ${\bar{p}}_{E}$ captures the overall performance impact, while the disparity between node groups is entirely characterized by the ratio of $\rho_{g}p_{g}^{2/\alpha }$. The constancy of the performance ratio between node groups, regardless of ν, aligns with expectations and stems directly from the assumption of a common TxPr. As a result, in RA-WMB with heterogeneous Tx power under a common TxPr, optimizing overall performance with respect to ν inherently maximizes the performance of each individual group.

### 3.3. Performance Loss Due to Heterogeneous Transmit Power Levels

This subsection examines the impact of heterogeneous Tx power configurations from an overall performance perspective. To achieve this, it quantitatively compares the performance with heterogeneous Tx power, as expressed in (8), to the performance when all nodes operate at a common Tx power equal to the average value $\bar{p}=E_{M}\left[M\right]$, as expressed in (7).

For a more precise comparison of the impact of heterogeneous Tx power configurations, the maximum performance achievable by adjusting the common TxPr for each configuration, namely $\max_{0<\nu <1}S\left(\Omega ,\nu \right)$ and $\max_{0<\nu <1}S_{E}\left(\bar{p},\nu \right)$, is examined. Accordingly, the *performance loss* (the term “loss” is used because this study analytically and empirically finds that S does not exceed $S_{E}$) due to heterogeneous Tx power configurations is measured by the *relative performance* of heterogeneous RA-WMB defined as

(16) $$\begin{array}{c} \begin{array}{c} \Theta \left(\Omega \right)≜\frac{\max_{0<\nu <1}S\left(\Omega ,\nu \right)}{\max_{0<\nu <1}S_{E}\left(\bar{p},\nu \right)}. \end{array} \end{array}$$

The following result provides an explicit demonstration of the properties of Θ.

**Corollary** **3.** *For a given* **Ω***, the relative performance, denoted by Θ(Ω), has the following properties when M is a random variable rather than a constant.*

*(i)*  *Θ(Ω) is equivalent to $\frac{\max_{0<\nu <1}S_{E}\left({\bar{p}}_{E},\nu \right)}{\max_{0<\nu <1}S_{E}\left(\bar{p},\nu \right)}$, where ${\bar{p}}_{E}=E_{M}{\left[M^{2/\alpha }\right]}^{\alpha /2}$ and $\bar{p}=E_{M}\left[M\right]$.*
*(ii)*  *Θ(Ω)<1. This indicates that the heterogeneous Tx power configuration leads to performance loss compared with the common Tx power configuration.*
*(iii)*  *In an interference-limited scenario where $\sigma^{2}\to 0$,*
(17) $$\begin{array}{c} \begin{array}{c} \lim_{\sigma^{2}\to 0}\Theta \left(\Omega \right)=1, \end{array} \end{array}$$
  *indicating that, asymptotically, there is no performance loss due to the heterogeneous Tx power configuration.*
*(iv)*  *In a coverage-limited scenario with no interference, modeled by K→∞,*
(18) $$\begin{array}{c} \begin{array}{c} \lim_{K\to \infty }\Theta \left(\Omega \right)=\frac{E_{M}\left[M^{2/\alpha }\right]}{E_{M}{\left[M\right]}^{2/\alpha }}<1, \end{array} \end{array}$$
  *implying that the heterogeneous Tx power configuration leads to performance loss, by a factor of $\frac{E_{M}\left[M^{2/\alpha }\right]}{E_{M}{\left[M\right]}^{2/\alpha }}$.*

**Proof.**  See Appendix C. □

From Corollary 3-(i), Θ can be expressed only in terms of $S_{E}$ values. As the common Tx power for $S_{E}$ increases, $S_{E}$ becomes progressively less sensitive to variations in common Tx power, eventually converging to the interference-limited case. This suggests that, as the equivalent Tx power ${\bar{p}}_{E}$ increases, Θ is expected to attain higher values, given an average Tx power of $\bar{p}$. Consequently, Θ reaches its minimum in the coverage-limited scenario, where the impact of Tx power is most significant, and gradually increases as the influence of interference grows, ultimately converging to one in the interference-limited environment. These findings will be validated through the numerical results presented in Section 5.

## 4. Design of Heterogeneous RA-WMB Operations

This section proposes a closed-form suboptimal TxPr to maximize the overall performance of heterogeneous RA-WMB and derives the optimal Tx power configuration to boost the performance of a specific node group, based on the results in Section 3.

### 4.1. Design of Transmission Probability

This subsection designs the TxPr ν to maximize the overall performance S for a given heterogeneous Tx power configuration Ω. Interestingly, as demonstrated in Corollary 2, the performance ratio among node groups operating at different Tx power levels remains constant under a given Ω. Consequently, maximizing the overall performance S is equivalent to maximizing the performance of any node group using a specific Tx power $p_{g}$. Based on this, the design of ν in this subsection aims to achieve optimal S for a given Ω, as follows.

(19) $$\begin{array}{c} \begin{array}{c} \nu^{*}\left(\Omega \right)≜\arg\max_{0<\nu <1}S\left(\Omega ,\nu \right) \end{array} \end{array}$$

As discussed in Section 3.1, since S(Ω,ν) can be expressed as $S_{E}\left({\bar{p}}_{E},\nu \right)$ from (9), it is evident that it shares the same properties as the optimal TxPr for RA-WMB with a common Tx power studied in [24]. The following result summarizes this straightforward conclusion for the sake of completeness.

**Remark** **1.** $\nu^{*}$ has the following properties:

*(i)*  It always holds that $0<\nu^{*}\leq \frac{1}{2}$.
*(ii)*  In an interference-limited scenario with $\sigma^{2}$, $\nu^{*}\to 0$.
*(iii)*  In a coverage-limited scenario with K→∞, $\nu^{*}=\frac{1}{2}$.

**Proof.**  The proof of (i) is similar to that in [24] (Proposition 3.1). In (8), for $\overset{˘}{\nu }>\frac{1}{2}$, $S(\Omega ,\overset{˘}{\nu })$ is less than $S(\Omega ,\frac{1}{2})$ because ν(1−ν) is maximized at $\nu =\frac{1}{2}$ and the integrand decreases as ν increases. (ii) and (iii) follow from Corollary 1. □

From Remark 1, the range of ν for maximizing S can be restricted to $0<\nu \leq \frac{1}{2}$. Further, based on empirical findings in [14,24], it is strongly conjectured that $S_{E}$ exhibits unimodality with respect to TxPr. Following these observations and (9), S is also expected to be unimodal in terms of TxPr. As a result, the optimal value $\nu^{*}$ can be obtained numerically using iterative methods such as the golden section method. To improve computational efficiency, however, the following result provides a closed-form suboptimal ν that approximates the optimal TxPr in (19) by maximizing the lower bound $\tilde{S}$ in (10) rather than S. This closed-form solution, unlike $\nu^{*}$ or the TxPr values proposed in [14,24], eliminates the need for iterative search methods.

**Theorem** **2.** *When $\tilde{\nu }\left(\Omega \right)≜\arg\max_{0<\nu \leq \frac{1}{2}}\tilde{S}\left(\Omega ,\nu \right)$,*

(20) $$\begin{array}{c} \begin{array}{c} \tilde{\nu }\left(\Omega \right)≜\frac{K{\left(\sigma^{2}\right)}^{2/\alpha }\tilde{\Delta }\left(\alpha \right)}{\pi \lambda E_{M}\left[M^{2/\alpha }\right]}+\frac{1}{2}-\sqrt{{\left(\frac{K{\left(\sigma^{2}\right)}^{2/\alpha }\tilde{\Delta }\left(\alpha \right)}{\pi \lambda E_{M}\left[M^{2/\alpha }\right]}\right)}^{2}+\frac{1}{4}}, \end{array} \end{array}$$

*where $\tilde{\Delta }\left(\alpha \right)$ is defined in Theorem 1.*

**Proof.**  See Appendix D. □

Meanwhile, ν to maximize $S_{E}\left(\bar{p},\nu \right)$ in (7) can be defined as

(21) $$\begin{array}{c} \begin{array}{c} \nu_{E}^{*}\left(\bar{p}\right)≜\arg\max_{0<\nu <1}S_{E}\left(\bar{p},\nu \right), \end{array} \end{array}$$

and, using the result in (20), a suboptimal ν for maximizing $S_{E}$ can also be designed as follows:

(22) $$\begin{array}{c} \begin{array}{c} {\tilde{\nu }}_{E}\left(\bar{p}\right)≜\tilde{\nu }\left(\left\{\bar{p},1\right\}\right)=\frac{K{\left(\sigma^{2}\right)}^{2/\alpha }\tilde{\Delta }\left(\alpha \right)}{\pi \lambda {\bar{p}}^{2/\alpha }}+\frac{1}{2}-\sqrt{{\left(\frac{K{\left(\sigma^{2}\right)}^{2/\alpha }\tilde{\Delta }\left(\alpha \right)}{\pi \lambda {\bar{p}}^{2/\alpha }}\right)}^{2}+\frac{1}{4}}. \end{array} \end{array}$$

### 4.2. Design of Heterogeneous Transmit Power Configuration

For a given $\bar{p}=E_{M}\left[M\right]$, one can consider a scenario that selectively boosts the performance of a specific node group. For example, under normal conditions, all nodes transmit with power $\bar{p}$. However, in urgent situations where a particular node group needs to transmit critical BMs, the performance of this single special group can be selectively enhanced, even if this slightly reduces overall performance. Although this scenario focuses on boosting a single group’s performance, it could be extended to sequentially enhance the performance of multiple groups as needed.

Accordingly, all node groups can be divided into a *primary group* and a *secondary group*, labeled as group 1 and group 2, respectively. For $\rho_{1},\rho_{2}>0$ and $p_{1},p_{2}\geq 0$, the corresponding heterogeneous Tx power configuration, Ω, is set as

(23) $$\begin{array}{c} \begin{array}{c} \Omega_{D}≜\left\{\left(p_{1},\rho_{1}\right),\left(p_{2},\rho_{2}\right)\right\}\;\mathrm{where}\;\rho_{1}+\rho_{2}=1,\rho_{1}p_{1}+\rho_{2}p_{2}=\bar{p},\mathrm{and}\;p_{1}>p_{2}. \end{array} \end{array}$$

As discussed in Section 3.3, the heterogeneous Tx power configuration may lead to a performance loss in overall performance. Therefore, a design approach is required to ensure that the relative performance measure, Θ in (16), does not become excessively low. In this context, this study imposes the constraint $\Theta (\Omega_{D})\geq \eta$, with a given 0<η<1, as a requirement for the heterogeneous Tx power configuration. Given the overall performance requirement for S, the heterogeneous Tx power configuration is designed to maximize the proportion of the primary group, expressed as $\frac{\rho_{1}p_{1}^{2/\alpha }}{\rho_{1}p_{1}^{2/\alpha }+\rho_{2}p_{2}^{2/\alpha }}$ from Corollary 2. Notably, maximizing $\frac{\rho_{1}p_{1}^{2/\alpha }}{\rho_{1}p_{1}^{2/\alpha }+\rho_{2}p_{2}^{2/\alpha }}$ is equivalent to maximizing the ratio $\frac{\rho_{1}p_{1}^{2/\alpha }}{\rho_{2}p_{2}^{2/\alpha }}$. As a result, this design can be formulated as follows:

(24a) $$\begin{array}{cc} \underset{p_{1},p_{2}\geq 0}{\mathrm{maximize}}\; & \frac{\rho_{1}p_{1}^{2/\alpha }}{\rho_{2}p_{2}^{2/\alpha }} \end{array}$$

(24b) $$\begin{array}{cc} \mathrm{subject}\;\mathrm{to}\; & \Theta (\Omega_{D})\geq \eta \end{array}$$

(24c) $$\begin{array}{cc} \; & \rho_{1}p_{1}+\rho_{2}p_{2}=\bar{p}, \end{array}$$

where $\rho_{1}+\rho_{2}=1$ for $\rho_{1},\rho_{2}>0$, η denotes the maximum allowable relative performance loss, where 0<η<1.

Before examining the optimal solution of Problem (24), this study first explores the relationship between $E_{M}\left[M^{2/\alpha }\right]$ for $\Omega =\Omega_{D}$ and the Tx power $p_{1}$ of the primary group, yielding the following result.

**Lemma** **1.** *When $\Omega =\Omega_{D}$ and $\rho_{1}$ and $\rho_{2}$ are given, $E_{M}\left[M^{2/\alpha }\right]$ decreases as $p_{1}$ increases.*

**Proof.**  See Appendix E. □

The following theorem provides the optimal values of $p_{1}$ and $p_{2}$ of Problem (24).

**Theorem** **3.** Let $p_{1}^{*}$ and $p_{2}^{*}$ denote the optimal solution of Problem (24). *And,*

(25) $$\begin{array}{c} \begin{array}{c} Q≜\max_{0<\nu \leq \frac{1}{2}}S_{E}\left(\bar{p},\nu \right). \end{array} \end{array}$$

*If $\max_{0<\nu \leq \frac{1}{2}}S\left(\rho_{1}^{\alpha /2}\bar{p},\nu \right)\geq \eta Q$,*

(26) $$\begin{array}{c} \begin{array}{c} p_{1}^{*}=\rho_{1}^{\alpha /2}\bar{p},\;p_{2}^{*}=0. \end{array} \end{array}$$

*Otherwise,*

(27) $$\begin{array}{c} \begin{array}{c} p_{1}^{*}=\tau ,\;p_{2}^{*}=\frac{\bar{p}-\rho_{1}\tau }{\rho_{2}}, \end{array} \end{array}$$

*where τ denotes the unique solution of $f_{t}\left(x\right)=\theta^{2/\alpha }$ when θ denotes a unique solution of $f_{q}\left(x\right)=\eta Q$. Here,*

(28) $$\begin{array}{c} \begin{array}{c} f_{q}\left(x\right)≜\max_{0<\nu \leq \frac{1}{2}}S_{E}\left(x,\nu \right)\;and\;f_{t}\left(x\right)≜\rho_{1}x^{2/\alpha }+\rho_{2}{\left(\frac{\bar{p}-\rho_{1}x}{\rho_{2}}\right)}^{2/\alpha }. \end{array} \end{array}$$

*and, as x increases, $f_{q}\left(x\right)$ monotonically increases while $f_{t}\left(x\right)$ monotonically decreases.*

**Proof.**  See Appendix F. □

From the monotonicity of $f_{q}\left(x\right)$ and $f_{t}\left(x\right)$, both θ and τ can be effectively obtained using the bisection method. However, in Theorem 3, the bisection method for determining θ requires calculating values of *Q* as expressed in (25), and each *Q* value involves applying the golden section method to derive $\max_{0<\nu \leq \frac{1}{2}}S_{E}\left(\bar{p},\nu \right)$. This approach has the drawback of requiring higher-complexity computations to achieve the optimal solution. To reduce this complexity, the computation of the optimal Tx power proposed in Theorem 3 may consider using the closed-form $\tilde{\nu }$ in (20) as an alternative to the optimal TxPr. This substitution also entails using the following $\tilde{\bar{Q}}$ in place of *Q*.

(29) $$\begin{array}{c} \begin{array}{c} \tilde{Q}≜\max_{0<\nu \leq \frac{1}{2}}S_{E}\left(\bar{p},\tilde{\nu }\right). \end{array} \end{array}$$

In Problem (24), this suboptimal approach ultimately replaces the constraint of $\Theta (\Omega_{D})\geq \eta$ in (24b), which is based on Θ using the optimal ν defined in (16), with the following constraint:

(30) $$\begin{array}{c} \begin{array}{c} \tilde{\Theta }\left(\Omega_{D}\right)\geq \eta \;\mathrm{where}\;\tilde{\Theta }\left(\Omega \right)≜\frac{S(\Omega ,\tilde{\nu })}{S_{E}\left(\bar{p},\tilde{\nu }\right)}. \end{array} \end{array}$$

As a result, using the constraint in (30) reduces computational complexity, eliminating the need for golden section methods to derive the optimal TxPr. The resulting suboptimal solution is denoted by $\left({\tilde{p}}_{1},{\tilde{p}}_{2}\right)$.

## 5. Numerical Results and Discussion

**Figure 2:**
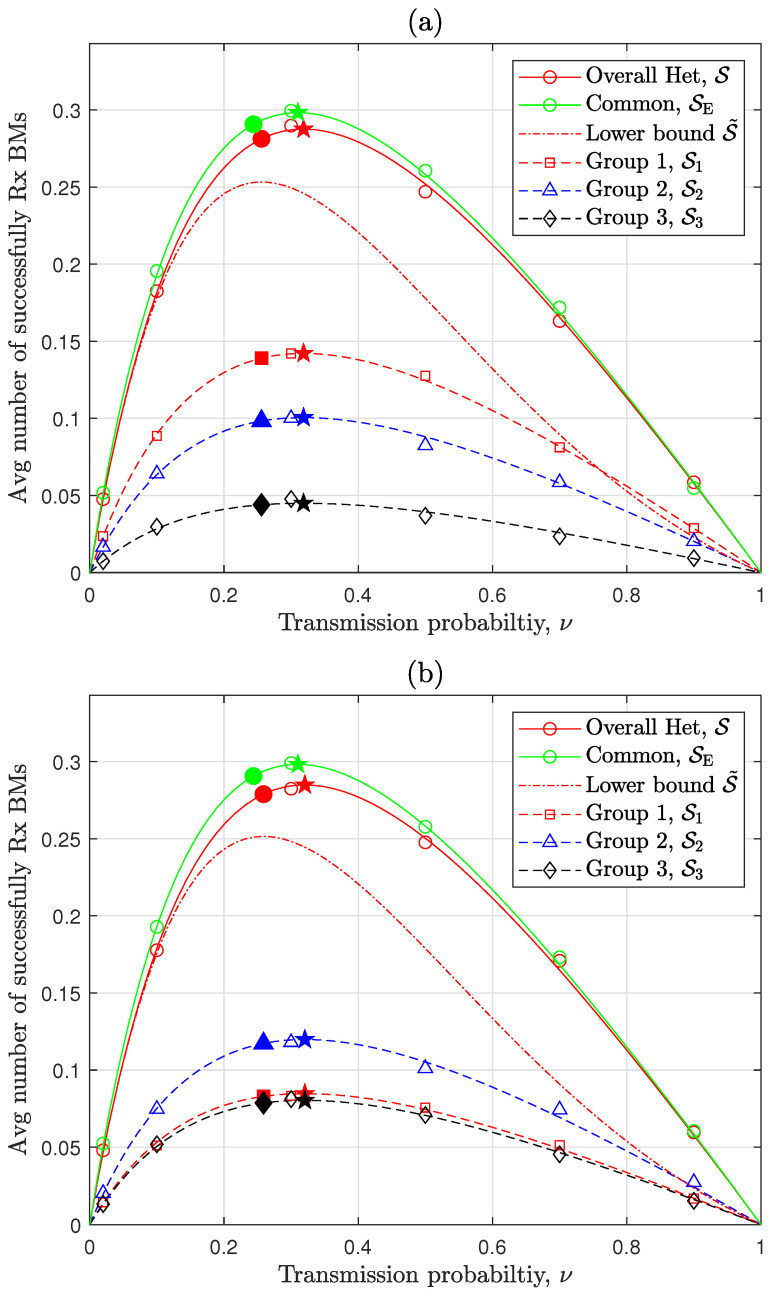
Overall and group-specific performances according to TxPr [lines: analysis, open symbols: simulation, solid pentagram: optimal TxPr $\nu^{*}$, other solid symbols: suboptimal TxPr $\tilde{\nu }$, $\lambda =20\;{\mathrm{km}}^{-2}$, α=4, $p_{g}$ ratio = 10:5:1]: (**a**) $\lambda_{g}$ ratio = 1:1:1. (**b**) $\lambda_{g}$ ratio = 1:2:3.

**Figure 3:**
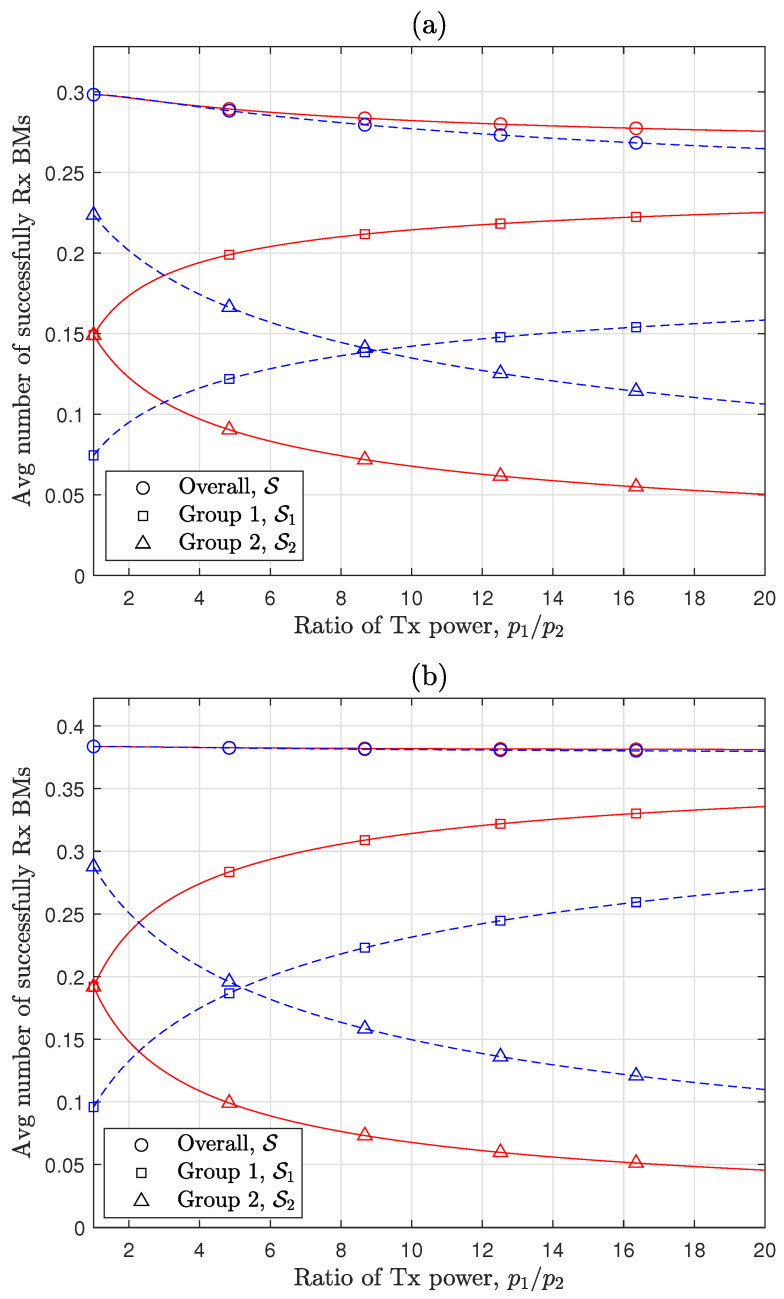
Performance variations by Tx power disparities among node groups [solid lines: $\lambda_{g}$ ratio = 1:1, dashed lines: $\lambda_{g}$ ratio = 1:3, $\lambda =20\;{\mathrm{km}}^{-2}$]: (**a**) α=4. (**b**) α=3.

**Figure 4:**
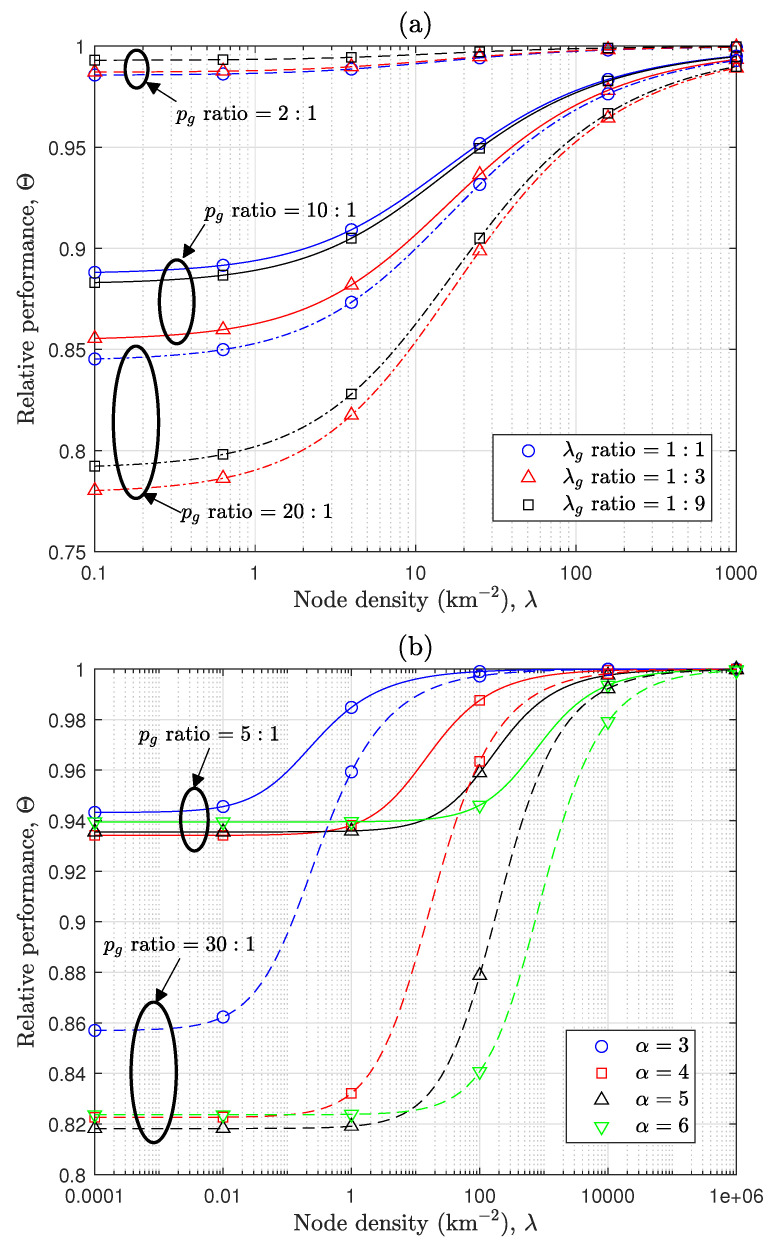
Comparative effects of various parameters on performance loss: (**a**) Effect of $\lambda_{g}$ ratio and $p_{g}$ ratio [α=4]. (**b**) Effect of $p_{g}$ ratio and PLE [$\lambda_{g}$ ratio = 1:1].

**Figure 5:**
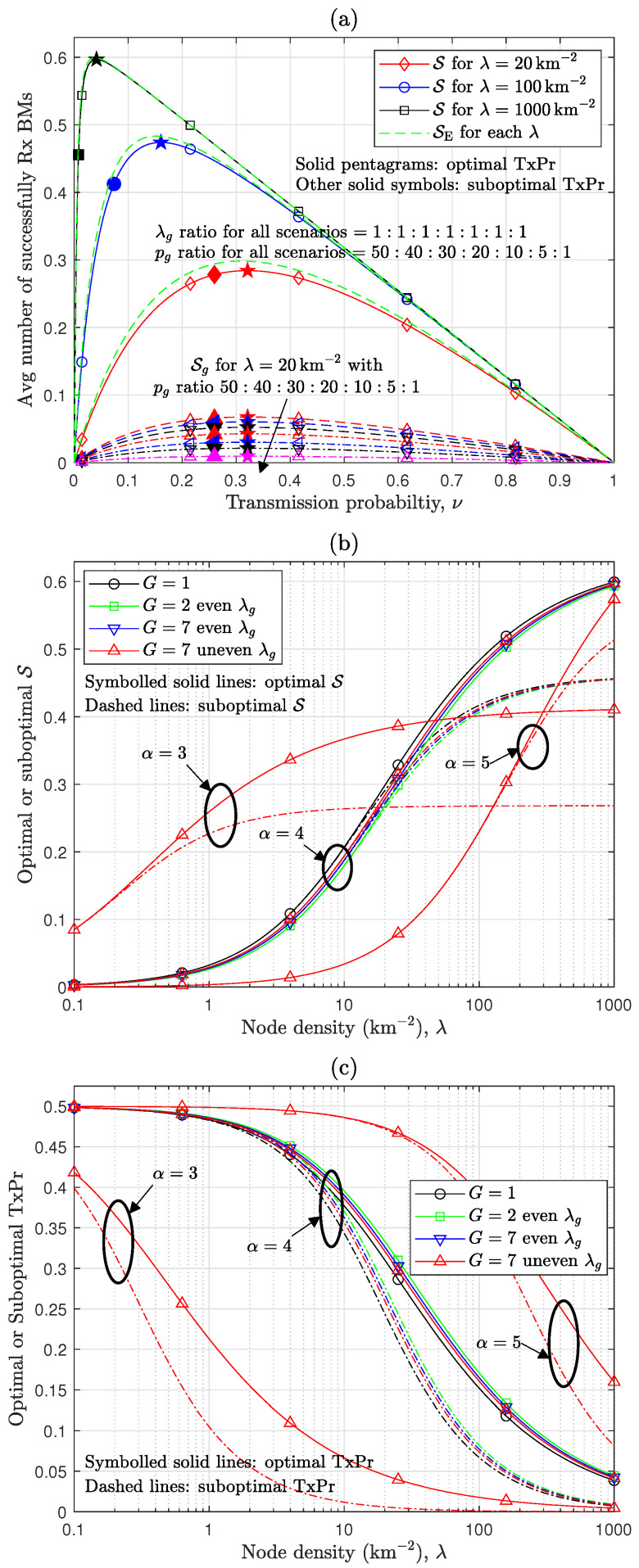
Comparison of optimal and suboptimal TxPr for various heterogeneous node group configurations: (**a**) S vs. ν [G=7]. (**b**) Optimal and suboptimal overall performances [$p_{g}$ ratio = 50:1 for G=2, $p_{g}$ ratio = 50:40:30:20:10:5:1 for G=7, $\lambda_{g}$ ratio = 1:2:3:4:6:8:10 for uneven $\lambda_{g}$]. (**c**) Optimal and suboptimal TxPr values [the same $p_{g}$ and $\lambda_{g}$ as those for (**b**)].

**Figure 6:**
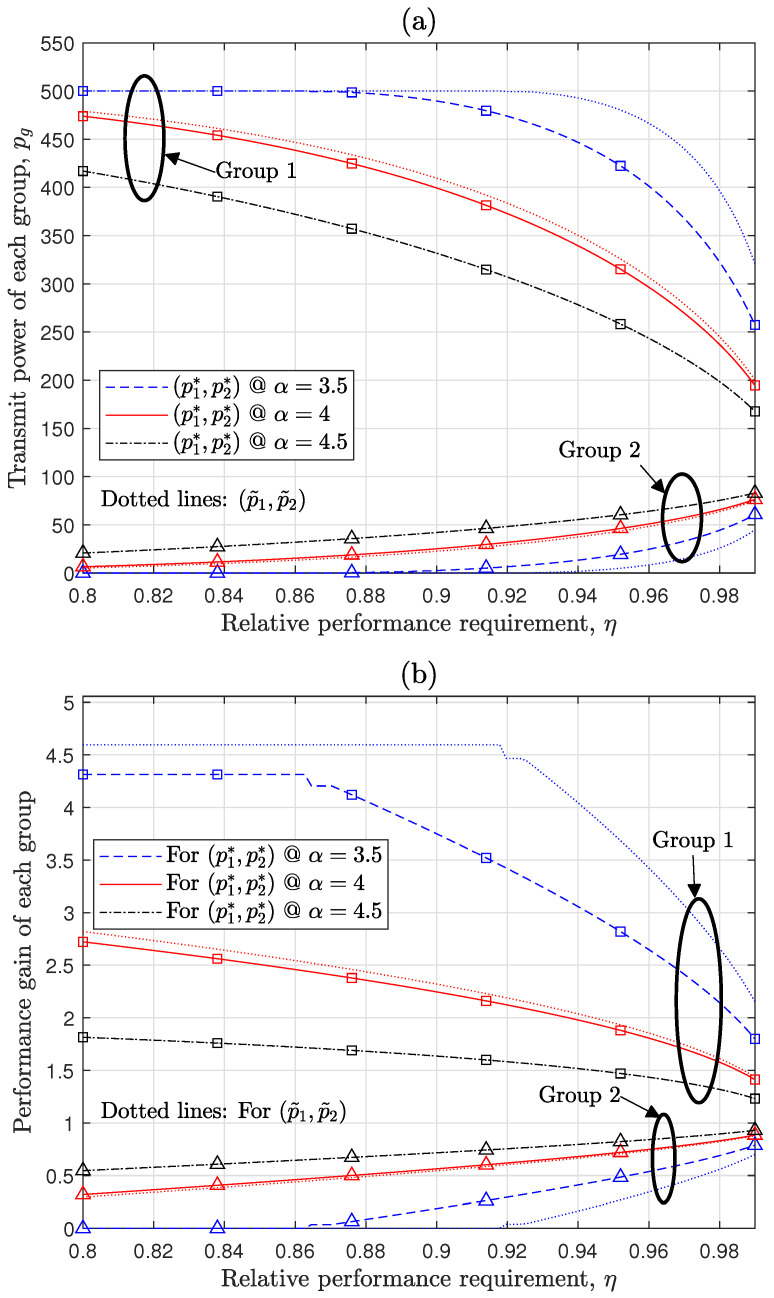
Performance prioritization through proposed heterogeneous Tx power configuration: [$\lambda =20\;{\mathrm{km}}^{-2}$, $\lambda_{g}$ ratio = 1:4] (**a**) Optimal and suboptimal Tx power values for each group. (**b**) Optimal and suboptimal performance gains for each group relative to common Tx power configuration.

This section presents a numerical analysis and discussion on the performance of the heterogeneous RA-WMB and the design approaches proposed in previous sections. The evaluation models are based on the system model described in Section 2.1, with the system parameters configured as specified in Table 1, unless stated otherwise. The impact of Tx power disparity on RA-WMB network performance, including both beneficial and adverse effects, is evaluated using the numerical results analyzed in Section 3, as displayed in Figure 2, Figure 3 and Figure 4. Additionally, the analytical results for the optimal and suboptimal values of TxPr and Tx power configurations, derived in Section 4 to optimize the performance of RA-WMB networks with Tx power disparity, are assessed through numerical results and presented in Figure 5 and Figure 6. The numerical computations for the analytical results derived in Section 3 and Section 4 involve closed-form expressions, numerical integration, bisection search, and golden section search. These numerical values are obtained using MATLAB R2023b. To validate the accuracy of the analysis for the baseline performance metric S, representing overall performance, and the individual group performance $S_{g}$ for nodes with distinct Tx powers, Figure 2 includes simulation results. For these simulations, the HPPP node distribution is realized by first generating the number of nodes within a given two-dimensional area as a Poisson random variable and then uniformly distributing the generated nodes across the area [15]. To ensure reliable SINR measurements, the performance is measured only for nodes located within a central region of radius $\frac{1}{12}$ of the simulation area’s total radius, thereby focusing on nodes receiving signals without edge effects. Each simulation is repeated 5000 times for every parameter configuration, and the average values are presented in Figure 2 as simulation results for S and $S_{g}$.

Figure 2 presents the overall performance of a heterogeneous RA-WMB network composed of three node groups with distinct Tx power levels and highlights the resulting performance differences among node groups due to these Tx power variations. The analytical results align closely with the simulation results. While S exhibits some deviations from its lower bounds $\tilde{S}$ in Theorem 1, the optimal TxPr value that maximizes S is closely approximated by $\tilde{\nu }$ in Theorem 2. As analyzed in Section 3.3, S under the heterogeneous Tx power configuration is slightly lower than $S_{E}$, where all nodes use the common Tx power $\bar{p}$. This performance difference will be further discussed in Figure 3 and Figure 4. Interestingly, Figure 2a,b exhibits similar overall performance despite variations in node density ratios under the current parameter settings. However, a notable performance difference is observed among the individual node groups. In Figure 2a, when the three node groups coexist in equal proportions, the ratio of $S_{g}$ values aligns precisely with the ratio derived in Corollary 2. And, S exhibits approximately a 3% performance loss compared with $S_{E}$, while $S_{1}$ shows a 1.5-fold increase relative to the common Tx power scenario (i.e., $\frac{1}{3}S_{E}$). In contrast, Figure 2b depicts the performance when heterogeneous node groups coexist in a 1:2:3 ratio, resulting in about a 4.7% decrease in S relative to $S_{E}$ but a 1.8-fold increase in $S_{1}$ compared with the common Tx power scenario (i.e., $\frac{1}{6}S_{E}$). Although $S_{1}$ remains smaller in absolute terms compared with $S_{2}$ due to its lower ratio given by $\frac{\lambda_{1}}{\lambda_{2}}=0.5$, its improvement over the common Tx power scenario is notable. As a result, a heterogeneous Tx power configuration can be beneficial for boosting specific group performance, even if it leads to a slight reduction in S. This trade-off between beneficial and adverse impacts varies with environmental factors, which will be further examined in Figure 6.

Figure 3 provides a more detailed examination of how heterogeneous Tx power configurations affect each group’s performance by focusing on two groups and examining performance variations with different Tx power ratios. The y-axis represents the optimal performance, i.e., $\max_{0<\nu \leq \frac{1}{2}}S$ and $\max_{0<\nu \leq \frac{1}{2}}S_{g}$, indicating the maximum achievable performance at each Tx power ratio. At $p_{1}/p_{2}=1$, the value at the intersection with the y-axis corresponds to the baseline performance of each group, depending only on their density ratio, based on $\max_{0<\nu \leq \frac{1}{2}}S_{E}$ achieved under a common Tx power configuration. As expected in previous sections, the performance gap between the two groups widens as the disparity between $p_{1}$ and $p_{2}$ increases. This observation clearly supports that, for a given $\bar{p}$, $E_{M}\left[M^{2/\alpha }\right]$ decreases as $p_{1}$ increases, as demonstrated in Lemma 1, resulting in a reduction in S according to (9). Moreover, comparing Figure 3a,b reveals that this loss intensifies with larger PLE values. For group-specific performance, with a node density ratio of 1:3, achieving double the performance of the common Tx power baseline (i.e., $\frac{1}{4}S_{E}$) for group 1 requires a $p_{1}/p_{2}$ configuration of approximately 6 when α=3 and 14 when α=4. This increased sensitivity to $p_{1}/p_{2}$ at lower PLEs is further highlighted by the intersection points of the $S_{g}$ values for both groups with a $\lambda_{g}$ ratio of 1:3, occurring at around $p_{1}/p_{2}=9$ for α=4 and $p_{1}/p_{2}=5.2$ for α=3. Consequently, while overall performance loss is more sensitive to $p_{1}/p_{2}$ at higher PLE values, performance differentiation between groups is more responsive to $p_{1}/p_{2}$ at lower PLE values. These findings emphasize the substantial impact of node density ratio, Tx power ratio, and wireless channel conditions on heterogeneous RA-WMB network performance.

Figure 4 examines the performance loss associated with heterogeneous Tx power configurations through the relative performance of Θ defined in (16) across various scenarios. To ensure clear comparisons, the number of groups is limited to two, and $\bar{p}$ is kept identical across all scenarios. As addressed in Section 3.3, Figure 3 presents that Θ in all scenarios achieves its minimum value in the coverage-limited case, determined by $\frac{E_{M}\left[M^{2/\alpha }\right]}{E_{M}{\left[M\right]}^{2/\alpha }}$, and gradually converges to one as the total node density increases. For a fixed $\bar{p}$, the value of Θ is ultimately governed by $E_{M}\left[M^{2/\alpha }\right]$, which is determined by a combination of the $p_{g}$ ratio, $\lambda_{g}$ ratio, and PLE. To evaluate their individual effects, Figure 3a examines the impact of $p_{g}$ and $\lambda_{g}$ ratios while keeping α fixed at four. The value of $E_{M}\left[M^{2/\alpha }\right]$ is expected to decrease as a difference in $p_{g}$ increases, provided that $\lambda_{g}p_{g}^{2/\alpha }$ does not become excessively dominant for one group. This is because, if the $\lambda_{g}p_{g}^{2/\alpha }$ value of one node group overwhelms that of the others, the network performance approaches that of a single dominant group, causing Θ to become closer to one. In fact, when two node groups coexist with a fixed $\lambda_{g}$ ratio, it has been mathematically proven in Lemma 1 that an increase in the difference between $p_{g}$ leads to a decrease in $E_{M}\left[M^{2/\alpha }\right]$. And, for fixed $p_{g}$ ratios, such as 10:1 or 20:1, Θ is smaller when the $\lambda_{g}$ ratio is 1:3 compared with 1:9. This is because, in the 1:9 case, the node group with smaller Tx power constitutes the majority, weakening the heterogeneous network characteristics. In contrast, when the $\lambda_{g}$ ratio is 1:1, Θ is larger than in the 1:3 case. This occurs because, with equal proportions of nodes, the higher Tx power nodes predominantly determine the overall network performance, thereby diminishing the heterogeneous network property. Ultimately, because $E_{M}\left[M^{2/\alpha }\right]$ is expressed as the sum of $\lambda_{g}p_{g}^{2/\alpha }$, both the $\lambda_{g}$ ratio and the $p_{g}$ ratio collectively influence Θ. As a result of this combined influence, it is observed that when the $p_{g}$ ratio is 2:1, the Θ value for a $\lambda_{g}$ ratio of 1:1 is slightly smaller than that for 1:3, which contrasts with the behavior observed for $p_{g}$ ratios of 10:1 or 20:1. Figure 4b illustrates the effect of PLE on Θ for different $p_{g}$ ratios, with the $\lambda_{g}$ ratio fixed at 1:1. As interference becomes dominant due to a high node density, as indicated in Corollary 3(ii), Θ always converges to one, regardless of the $\lambda_{g}$ ratio, $p_{g}$ ratio, or PLE. Smaller values of α lead to faster convergence, and when λ exceeds a certain value (e.g., $\lambda \geq 20\;{\mathrm{km}}^{-2}$), smaller PLE values result in larger Θ values. This phenomenon occurs because smaller values of α result in more dominant interference. As inferred from Corollary 3(ii), greater interference causes Θ to approach closer to one. In contrast, in coverage-limited scenarios with very small λ, Θ equals $\frac{E_{M}\left[M^{2/\alpha }\right]}{E_{M}{\left[M\right]}^{2/\alpha }}$, which is influenced by the combined effects of the $\lambda_{g}$ ratio, $p_{g}$ ratio, and PLE. As a result, the results in Figure 4 demonstrate that Θ is influenced by a combination of the $\lambda_{g}$ ratio, $p_{g}$ ratio, and PLE, and notably, an increase in the $p_{g}$ ratio exhibits a clear tendency to reduce Θ.

Figure 5 compares the performance of the optimal and suboptimal TxPr in environments where multiple node groups coexist with distinct Tx power levels. In Figure 5a, the case with $\lambda =20\;{\mathrm{km}}^{-2}$ extends the results of Figure 2 for G=3 to G=7, where seven node groups coexist with even ratios. The ratio of $S_{g}$ among node groups corresponds precisely to the ratio of $p_{g}^{2/\alpha }$ for a given TxPr, as demonstrated in Corollary 2. Because all node groups use a common TxPr in this study, the optimal TxPr $\nu^{*}$ that maximizes the overall performance also maximizes the individual $S_{g}$. It is observed that the suboptimal TxPr $\tilde{\nu }$ closely approximates the optimal TxPr $\nu^{*}$ for $\lambda =20\;{\mathrm{km}}^{-2}$. However, as shown in Figure 6a, an increase in λ and the resulting intensification of interference effects cause the ascending side of S within the TxPr interval below $\nu^{*}$ to exhibit a steeper change compared with the descending side above $\nu^{*}$. Thus, even a slight difference between $\nu^{*}$ and $\tilde{\nu }$ can lead to a notable increase in the gap between $S(\Omega ,\nu^{*})$ (referred to as ‘the optimal S’) and $S(\Omega ,\tilde{\nu })$ (referred to as ‘the suboptimal S’) for very high λ (e.g., $\lambda =1000,{\mathrm{km}}^{-2}$), where interference becomes dominant and $\nu^{*}$ approaches zero. To further examine the performance differences between the optimal and suboptimal TxPr, Figure 5b,c exhibit the optimal and suboptimal S as well as the corresponding $\nu^{*}$ and $\tilde{\nu }$ for various values of λ. As discussed in Figure 5a, the suboptimal S closely approximates the optimal S unless interference becomes excessively high due to a large λ. However, the performance gap increases significantly when λ reaches extremely high levels. In this context, the threshold for what constitutes a high λ may depend on the PLE value. The results indicate that across various PLE values, the performance gap between the optimal and suboptimal S remains below about 10% under conditions where $\nu^{*}\geq 0.2$. For practical RA-WMB operation, avoiding excessive congestion is essential to maintaining acceptable latency for BM transmissions. Although this numerical evaluation sets the number of orthogonal RBs (denoted by *K*) to one for more intuitive examination, increasing *K* (e.g., configuring *K* such that $\nu^{*}\geq 0.2$) can keep the node density per RB at a manageable level, preventing very low $\nu^{*}$ values that result in high latency. Thus, the closed-form suboptimal TxPr $\tilde{\nu }$ proposed in Theorem 2 provides a reasonable approximation of $\nu^{*}$ with a small performance gap in environments, e.g., where $\nu^{*}\geq 0.2$ is achievable. Meanwhile, Figure 5b,c compares the performance across different node group sizes, including G=1, G=2, and G=7, under the condition α=4. In all scenarios, $\bar{p}$ remains identical. For G=2, the $p_{g}$ ratio is 50:1, whereas for G=7, it is further divided into 50:40:30:20:10:5:1, maintaining the same maximum-to-minimum ratio as in G=2. As discussed in Section 3.3, the performance loss resulting from heterogeneous Tx power causes the optimal S for G=2 and G=7 to be lower than that for G=1. Notably, when the $\lambda_{g}$ ratio is even, G=2 shows the lowest optimal S among the cases. This is because the more gradual differences in Tx power among node groups in G=7 mitigate the disparity observed in G=2. Additionally, for G=7 in an uneven $\lambda_{g}$ ratio environment of 1:2:3:4:6:8:10, certain node groups with specific Tx power values, such as the first and second smallest Tx power groups, form the majority, collectively accounting for over 50% of the total. This reduces the performance disparity among node groups, resulting in less performance loss compared with the even $\lambda_{g}$ scenario.

Figure 6 evaluates the performance of the heterogeneous Tx power configuration proposed in Section 4.2 to maximize the primary group’s performance under a given performance loss requirement. In this figure, the primary and secondary groups are denoted by ‘Group 1’ and ‘Group 2’, respectively. The results present the optimal Tx power configuration for each group as a function of the relative performance loss requirement η in Figure 6a and the resulting performance gain of each group compared with the common Tx power case in Figure 6b. The optimal solution to Problem (24), based on Theorem 3, aims to maximize the primary group’s Tx power $p_{1}$ (or equivalently minimize $p_{2}$) while satisfying the constraint of $\Theta (\Omega_{D})\geq \eta$ in (24b) and the requirement of $\bar{p}$ in (24c). As shown in Figure 6a, assuming (24c) is satisfied, the constraint (24b) is violated when $p_{1}>p_{1}^{*}$ or $p_{2}<p_{2}^{*}$, but it is satisfied when $p_{1}<p_{1}^{*}$ and $p_{2}>p_{2}^{*}$. In this context, Figure 6a effectively illustrates the boundaries of $p_{1}^{*}$ and $p_{2}^{*}$ for a given η, accurately representing the optimal configuration. As η increases, the performance loss constraint becomes stricter, limiting the primary group’s performance improvement. This trend is evident as the primary group’s Tx power decreases in Figure 6a and its performance gain diminishes in Figure 6b. Consistent with Figure 3, increased PLE leads to higher performance loss in interference-dominant scenarios, as reflected in the reduction in the primary group’s optimal Tx power and performance gain in Figure 6a,b. For α=3.5 and η≤0.86, the primary group transmits at its maximum power $\frac{\bar{p}}{\rho_{1}}$, while the secondary group remains inactive, satisfying Θ≥η without violating (24b). When the suboptimal $\tilde{\Theta }$, defined in (30), is used instead of Θ, the suboptimal Tx power pair $\left({\tilde{p}}_{1},{\tilde{p}}_{2}\right)$ closely approximates $\left(p_{1}^{*},p_{2}^{*}\right)$, especially as PLE increases. This alignment is due to the suboptimal TxPr’s ability to better approximate $\nu^{*}$ in low or moderate interference environments, as addressed in the previous paragraph describing Figure 5. Using the closed-form $\tilde{\nu }$ eliminates the need for iterative algorithms like the Golden section search, significantly reducing the complexity of designing heterogeneous Tx power configurations. While $\left({\tilde{p}}_{1},{\tilde{p}}_{2}\right)$ satisfies the constraint (30) for the suboptimal problem, it does not satisfy the original constraint (24b) in Problem (24) because ${\tilde{p}}_{1}$ exceeds $p_{1}^{*}$ and ${\tilde{p}}_{2}$ is smaller than $p_{2}^{*}$. Rather than interpreting this as a violation of the original problem, it is more appropriate to view the Tx power configuration as being designed within the framework of Problem (24), with the constraint transformed from (24b) to (30).

## 6. Conclusions

This paper analytically investigated the network-level performance of a heterogeneous RA-WMB composed of nodes operating at distinct transmit (Tx) power levels under a common transmission probability (TxPr). In particular, by expressing the performance of heterogeneous RA-WMB in terms of an equivalent RA-WMB with a common Tx power, the analysis enabled precise comparisons of heterogeneous and common Tx power configurations. Based on these findings, the study demonstrated that heterogeneous RA-WMB suffers its worst performance loss relative to common Tx power RA-WMB in coverage-limited scenarios, with this loss diminishing as interference intensifies and ultimately approaching the performance of common Tx power configurations. Numerical results also presented that the path loss exponent has a significant impact on overall performance loss, while performance differences among node groups are strongly affected by the Tx power ratio between groups even when overall performance loss is minor. Considering that larger Tx power differences among node groups increase performance loss, an optimization problem was formulated to prioritize the WMB performance of a specific group without exceeding acceptable performance loss, and the optimal Tx power configuration is derived. To avoid repeated iterative calculations of the optimal TxPr in this optimization, a closed-form suboptimal TxPr was proposed as an efficient alternative. Future research could be extended to a more generalized heterogeneous RA-WMB, in which both Tx power and TxPr are distinct across nodes, exploring the joint optimization of Tx power and TxPr.

## Figures and Tables

**Table 1:** Notations and their description for heterogeneous RA-WMB.

Notations	Descriptions	Expressions or Values
*A*	Path loss gain at a unit distance	−44.48dB @ 4GHz & 1m
*G*	Number of possible Tx power levels	Not limited, 2 or 3
$h_{i}$	Rayleigh fading gain from node *i* to the typical node	Unit mean
$I_{ij}$	Interfering status of node *j* with respect to node *i*	In (1)
$I(X_{i})$	Interference affecting the BM transmitted by node *i*	In (1)
*K*	Number of orthogonal RBs	1, possible to be more
M	Set of possible Tx power levels	$\left\{p_{1},\cdots ,p_{G}\right\}$
*M*	Random variable for collectively representing $m_{i}$’s	-
$m_{i}$	Mark for Tx power of node *i*	-
$\bar{p}$, $p_{g}$	Average Tx power, Tx power of node group *g*	$\bar{p}=100\;\mathrm{mW}$
${\bar{p}}_{E}$	Equivalent Tx power for expressing S in $S_{E}$	In (9)
S	Average number of successfully received BMs from all nodes per node	In (8)
$\tilde{S}$	Lower bound of S	In (11)
$S_{E}$	S of RA-WMB with common Tx power	In (7)
$S_{g}$	S of node group *g*	In (15)
$T_{i}$	Transceiving status of node *i*	In (1)
$X_{i}$	Node *i* or its location	-
α	Path loss exponent	3 to 6
Δ(α), $\tilde{\Delta }\left(\alpha \right)$	Constants depending only on α defined in Theorem 1	In Theorem 1
η	maximum allowable relative performance loss in Problem 24	0<η<1
Θ	Relative performance due to heterogeneous Tx power	In (16)
$\tilde{\Theta }$	Altenative to Θ	In (30)
λ, $\lambda_{g}$	Total node density, node density of node group *g*	$\lambda =20\;{\mathrm{km}}^{-2}$
ν	Transmission probability (TxPr)	0<ν<1
$\nu^{*}$, $\tilde{\nu }$	Optimal and suboptimal TxPr’s for maximizing S	In
$\nu_{E}^{*}$, ${\tilde{\nu }}_{E}$	Optimal and suboptimal TxPr’s for maximizing $S_{E}$	In (19) and (20)
Ξ	SINR of BM received by the typical node	In (1)
ξ	SINR threshold for successful BM receiving	0dB
$\rho_{g}$	Portion of nodes with Tx power $p_{g}$	In (14)
$\sigma^{2}$	Noise power normalized by *A*	−73.95dBm for 360kHz RB (−174dBm/Hz PSD)
Φ, $\hat{\Phi }$	Set of nodes, marked point process for node and Tx power	-
Ω	Set of $\left(p_{g},\rho_{g}\right)$	In (2)
$\Omega_{D}$	Ω for two groups	In (23)

## Data Availability

Data are contained within the article.

## Abbreviations

The following abbreviations are used in this manuscript:

3D	Three dimensional
5G	Fifth generation
6G	Sixth generation
BLE	Bluetooth Low Energy
BM	Broadcast message
CSMA	Carrier-sensing multiple access
HPPP	Homogeneous Poisson point process
iid	Independent and identically distributed
IoT	Internet of things
MPR	Multipacket reception
PDF	Probability density function
PLE	Path loss exponent
PPP	Poisson point process
PSD	Power spectral density
QoS	Quality of service
RA-WMB	Random access based wireless mutual broadcast
RB	Resource block
RSU	roadside unit
Rx	Received
SIR	Signal to interference ratio
SINR	Signal to interference plus noise ratio
Tx	Transmit
TxPr	Transmission probability
UAV	Unmanned aerial vehicle
V2V	Vehicle to vehicle
WMB	Wireless mutual broadcast

## Appendix A

**Proof of** **Theorem 1.** S(Ω,ν) in (3) is expressed as

(A1) $$\begin{array}{c} \begin{array}{cc} S(\Omega ,\nu )\; & \overset{\left(a\right)}{=}\nu \left(1-\nu \right)E\left[\sum_{i:\left(X_{i},m_{i}\right)\in \hat{\Phi }\setminus \left\{\left(X_{o},m_{o}\right)\right\}}\;1\left[\Xi (X_{i},m_{i})>\xi \right]\right]\\ \; & \overset{\left(b\right)}{=}\lambda \nu \left(1-\nu \right)E_{M}\left[\int_{R^{2}}P\left[h>\frac{{\left|x\right|}^{\alpha }\xi \left(\sigma^{2}+I\left(x\right)\right)}{M}\right]dx\right]\\ \; & \overset{\left(c\right)}{=}2\pi \lambda \nu \left(1-\nu \right)E_{M}\left[\int_{0}^{\infty }e^{-\frac{\xi \sigma^{2}}{M}r^{\alpha }}L_{I}\left(\frac{r^{\alpha }\xi }{M}\right)rdr\right]\\ \; & \overset{\left(d\right)}{=}2\pi \lambda \nu \left(1-\nu \right)E_{M}\left[\int_{0}^{\infty }e^{-\frac{\xi \sigma^{2}}{M}r^{\alpha }-\pi \frac{\lambda \nu \xi^{2/\alpha }}{K\Delta (\alpha )}\frac{E_{L}\left[L^{2/\alpha }\right]}{M^{2/\alpha }}r^{2}}rdr\right], \end{array} \end{array}$$

where (a) follows from (3) and the definition of ν, (b) follows from Campbell’s theorem for stationary iid marked point processes (PP) [15] (Corollary 7.4), which states that $E\left[\sum_{\left(x,m\right)\in \hat{\Phi }}f\left(x,m\right)\right]=\lambda E_{M}\left[\int_{R^{2}}f\left(x,M\right)dx\right]$, with random variable *M* collectively representing the iid mark $m_{i}$’s, *h* denotes a random variable denoting the Rayleigh fading gain, (c) follows from r≜|x| and $L_{I}\left(s\right)≜E\left[e^{-sI}\right]$, and (d) follows from $E\left[e^{-sI}\right]=E\left[\prod_{\left(X_{j},m_{j}\right)\in {\hat{\Phi }}^{\nu /K}}\frac{1}{1+sm_{j}{\left|X_{j}\right|}^{-\alpha }}\right]=e^{-2\pi \frac{\lambda \nu }{K}U\left(s\right)}$ from the probability generating functional of HPPP [15], when ${\hat{\Phi }}^{\nu /K}$ denotes the independent thinning of $\hat{\Phi }$ with retention probability $\frac{\nu }{K}$ capturing the randomness of Tx nodes’ RB selection. Here, when *L* denotes a random variable representing iid $m_{j}$’s,

(A2) $$\begin{array}{c} \begin{array}{cc} \; & {\left.U\left(s\right)\right|}_{s=\frac{r^{\alpha }\xi }{M}}≜{\left.E_{L}\left[\int_{0}^{\infty }\frac{sLt^{-\alpha }}{1+sLt^{-\alpha }}tdt\right]\right|}_{s=\frac{r^{\alpha }\xi }{M}}=E_{L}\left[\int_{0}^{\infty }\frac{1}{1+\frac{M}{L\xi }{\left(\frac{t}{r}\right)}^{\alpha }}tdt\right]\\ \; & \overset{\left(a\right)}{=}E_{L}\left[\frac{1}{2}{\left(\frac{\xi }{M}\right)}^{2/\alpha }r^{2}L^{2/\alpha }\int_{0}^{\infty }\frac{1}{1+v^{\alpha /2}}dv\right]=\frac{1}{2}{\left(\frac{\xi }{M}\right)}^{2/\alpha }r^{2}E_{L}\left[L^{2/\alpha }\right]\int_{0}^{\infty }\frac{1}{1+v^{\alpha /2}}dv\\ \; & =\frac{1}{2}\frac{\xi^{2/\alpha }}{\Delta (\alpha )}\frac{E_{L}\left[L^{2/\alpha }\right]}{M^{2/\alpha }}r^{2}, \end{array} \end{array}$$

where (a) follows from a change in variable $v≜{\left(\frac{M}{L\xi }\right)}^{\frac{2}{\alpha }}{\left(\frac{t}{r}\right)}^{2}$. In (A1),

(A3) $$\begin{array}{c} \begin{array}{cc} \; & E_{M}\left[\int_{0}^{\infty }e^{-\frac{\xi \sigma^{2}}{M}r^{\alpha }-\pi \frac{\lambda \nu \xi^{2/\alpha }}{K\Delta (\alpha )}\frac{E_{L}\left[L^{2/\alpha }\right]}{M^{2/\alpha }}r^{2}}rdr\right]\\ \; & \overset{\left(a\right)}{=}\frac{1}{2}E_{M}\left[M^{2/\alpha }\int_{0}^{\infty }e^{-\xi \sigma^{2}u^{\alpha /2}-\pi \frac{\lambda \nu \xi^{2/\alpha }}{K\Delta (\alpha )}E_{L}\left[L^{2/\alpha }\right]u}du\right]\\ \; & =\frac{1}{2}E_{M}\left[M^{2/\alpha }\right]\int_{0}^{\infty }e^{-\xi \sigma^{2}u^{\alpha /2}-\pi \frac{\lambda \nu \xi^{2/\alpha }}{K\Delta (\alpha )}E_{L}\left[L^{2/\alpha }\right]u}du, \end{array} \end{array}$$

where (a) follows from the change of variable $u≜\frac{r^{2}}{M^{2/\alpha }}$. Finally, from $E_{M}\left[M^{2/\alpha }\right]=E_{L}\left[L^{2/\alpha }\right]$, (8) is obtained. On the other hand, the expression for $S_{E}$ with $M=\bar{p}$, which is defined in (7), can be obtained by replacing $E_{M}\left[M^{2/\alpha }\right]$ with ${\bar{p}}^{2/\alpha }$ in (8) because $M=\bar{p}$, and it results in

(A4) $$\begin{array}{c} \begin{array}{cc} \; & S_{E}\left(\bar{p},\nu \right)=\pi \lambda \nu \left(1-\nu \right){\bar{p}}^{2/\alpha }\int_{0}^{\infty }e^{-\xi \sigma^{2}u^{\alpha /2}-\pi \frac{\lambda \xi^{2/\alpha }}{K\Delta (\alpha )}{\bar{p}}^{2/\alpha }\nu u}du, \end{array} \end{array}$$

which is equal to the expression in [24] (Lemma 2.1). Conversely, S in (8) can be reexpressed by substituting $E_{M}{\left[M^{2/\alpha }\right]}^{2/\alpha }$ for $\bar{p}$ in (A4). This indicates that S can be equivalently represented by $S_{E}$. Therefore, (9) is derived. For the lower bound of S, when considering random variable *U* whose PDF is $F_{U}\left(u\right)=\frac{1}{\phi (\alpha )}e^{-\xi \sigma^{2}u^{\alpha /2}}$, where $\phi \left(\alpha \right)≜\int_{0}^{\infty }e^{-\xi \sigma^{2}u^{\alpha /2}}du={\left(\frac{1}{\xi \sigma^{2}}\right)}^{2/\alpha }\Gamma \left(1+\frac{2}{\alpha }\right)$, S in (8) can be reexpressed as follows:

(A5) $$\begin{array}{c} \begin{array}{cc} S & =\pi \lambda \nu \left(1-\nu \right)E_{M}\left[M^{2/\alpha }\right]\phi \left(\alpha \right)E_{U}\left[e^{-\pi \frac{\lambda \nu \xi^{2/\alpha }}{K\Delta (\alpha )}E_{M}\left[M^{2/\alpha }\right]U}\right]\\ \; & \overset{\left(a\right)}{<}\pi \lambda \nu \left(1-\nu \right)E_{M}\left[M^{2/\alpha }\right]\phi \left(\alpha \right)e^{-\pi \frac{\lambda \nu \xi^{2/\alpha }}{K\Delta (\alpha )}E_{M}\left[M^{2/\alpha }\right]E_{U}\left[U\right]}\\ \; & \overset{\left(b\right)}{=}\tilde{S}, \end{array} \end{array}$$

where (a) follows from the Jensen’s inequality, and (b) follows from $E_{U}\left[U\right]=\int_{0}^{\infty }uF_{U}\left(u\right)du$$={\left(\frac{1}{\xi \sigma^{2}}\right)}^{2/\alpha }\frac{\Gamma (4/\alpha )}{\Gamma (2/\alpha )}$ and $\phi \left(\alpha \right)={\left(\frac{1}{\xi \sigma^{2}}\right)}^{2/\alpha }\Gamma \left(1+\frac{2}{\alpha }\right)$. As a result, (10) holds. □

## Appendix B

**Proof of** **Corollary 2.** In (A1), by considering only the BMs successfully received from $\left(X_{i},m_{i}\right)\in \hat{\Phi }$ where $m_{i}=p_{g}$ and fixing $m_{i}$ as $p_{g}$ in $\Xi (X_{i},m_{i})$, $S_{g}\left(\Omega ,\nu \right)$ in (4) can be expressed with ${\hat{\Phi }}^{\rho_{g}}$ independently thinning $\hat{\Phi }$ with retention probability $\rho_{g}$, as follows.

(A6) $$\begin{array}{c} \begin{array}{cc} S_{g}\left(\Omega ,\nu \right)\; & =E\left[1\left[T_{0}=0\right]\;\sum_{i:\left(X_{i},m_{i}\right)\in {\hat{\Phi }}^{\rho_{g}}\setminus \left\{\left(X_{o},m_{o}\right)\right\}}\;1\left[T_{i}=1,m_{i}=p_{g},\Xi \left(X_{i},m_{i}\right)>\xi \right]\right]\\ \; & \overset{\left(a\right)}{=}E\left[1\left[T_{0}=0\right]\;\sum_{i:\left(X_{i},m_{i}\right)\in {\hat{\Phi }}^{\rho_{g}}\setminus \left\{\left(X_{o},m_{o}\right)\right\}}\;1\left[T_{i}=1,\Xi \left(X_{i},p_{g}\right)>\xi \right]\right]\\ \; & \overset{\left(b\right)}{=}\pi \lambda \nu \left(1-\nu \right)\rho_{g}p_{g}^{2/\alpha }\int_{0}^{\infty }e^{-\xi \sigma^{2}u^{\alpha /2}-\pi \frac{\lambda \nu \xi^{2/\alpha }}{K\Delta (\alpha )}E_{L}\left[L^{2/\alpha }\right]u}du\\ \; & \overset{\left(c\right)}{=}\frac{\rho_{g}p_{g}^{2/\alpha }}{E_{M}\left[M^{2/\alpha }\right]}S\left(\Omega ,\nu \right)\overset{\left(d\right)}{=}\frac{\rho_{g}p_{g}^{2/\alpha }}{E_{M}\left[M^{2/\alpha }\right]}S_{E}\left({\bar{p}}_{E},\nu \right), \end{array} \end{array}$$

where (a) follows from the fact that $m_{i}$ is set at constant $p_{g}$ rather than a random variable, (b) follows from the derivation similar to that of (8), and (c) and (d) follow directly from the expressions of S in (8) and (9). As a result, the ratio between $\rho_{g}p_{g}^{2/\alpha }$ values reflects the ratio of $S_{g}$ values. □

## Appendix C

**Proof of Corollary** **3.**

(i) The result in (i) follows from (9) and the definition of Θ in (16).
(ii) By Jensen’s inequality, $E_{M}\left[M^{2/\alpha }\right]<E_{M}{\left[M\right]}^{2/\alpha }$ holds due to the concavity of $M^{2/\alpha }$ for α>2 with respect to *M*. Since $S_{E}$ is obviously an increasing function of the common Tx power, $\max_{0<\nu <1}S_{E}$ is also an increasing function of the common Tx power. The result of (i) and these observations establish that $\max_{0<\nu <1}S\left(\Omega ,\nu \right)=\max_{0<\nu <1}S_{E}\left({\bar{p}}_{E},\nu \right)<\max_{0<\nu <1}S_{E}\left(\bar{p},\nu \right)$, which implies that Θ<1.
(iii) From (9) and (12), both $\lim_{\sigma^{2}\to 0}S$ and $\lim_{\sigma^{2}\to 0}S_{E}$ approach their upper bounds as ν→0, with these values being equal; thus, (17) is established.
(iv) From (9) and (13), both $\lim_{K\to \infty }S$ and $\lim_{K\to \infty }S_{E}$ are maximized at $\nu =\frac{1}{2}$. Thus, it holds that $\lim_{K\to \infty }\Theta \left(\Omega \right)=\frac{E_{M}\left[M^{2/\alpha }\right]}{E_{M}{\left[M\right]}^{2/\alpha }}$. Further, from the relation of $E_{M}{\left[M^{2/\alpha }\right]}^{\alpha /2}<E_{M}{\left[M\right]}^{2/\alpha }$ by Jensen’s inequality, $\lim_{K\to \infty }\Theta \left(\Omega \right)<1$.

□

## Appendix D

**Proof of Theorem** **2.** From (11), $\tilde{S}$ is log-concave with respect to $0<\nu \leq \frac{1}{2}$. Therefore, when expressing $\tilde{S}$ in a form of $C_{0}\nu \left(1-\nu \right)e^{-C_{1}\nu }$ for $C_{0},C_{1}>0$, $\tilde{\nu }$ meets that

(A7) $$\begin{array}{c} \begin{array}{c} \frac{\partial }{\partial \nu }\log\tilde{S}=\frac{1}{\nu }-\frac{1}{1-\nu }-C_{1}=0. \end{array} \end{array}$$

When $f\left(\nu \right)≜\nu^{2}-2\left(\frac{1}{2}+\frac{1}{C_{1}}\right)\nu +\frac{1}{C_{1}}$, f(ν) meets that $f^{\prime }\left(\frac{1}{2}+\frac{1}{C_{1}}\right)=0$, $f\left(0\right)=\frac{1}{C_{1}}>0$ and $f\left(\frac{1}{2}\right)=-\frac{1}{4}$. These properties imply that f(ν) has only a single zero for $0<\nu <\frac{1}{2}$ from graphical properties of a concave quadratic function; thus, $\tilde{\nu }=\frac{1}{C_{1}}+\frac{1}{2}-\sqrt{\frac{1}{C_{1}^{2}}+\frac{1}{4}}$ from f(ν)=0 for $0<\nu <\frac{1}{2}$. As a result, (20) holds, where $C_{1}=\frac{\pi \lambda }{K{\left(\sigma^{2}\right)}^{2/\alpha }\tilde{\Delta }\left(\alpha \right)}E_{M}\left[M^{2/\alpha }\right]$. □

## Appendix E

**Proof of Lemma** **1.** For $\Omega =\Omega_{D}$, $E_{M}\left[M^{2/\alpha }\right]=\rho_{1}p_{1}^{2/\alpha }+\rho_{2}p_{2}^{2/\alpha }$. Also, $\bar{p}=\rho_{1}p_{1}+\rho_{2}p_{2}$ and the derivative of this equation with respect to $p_{1}$ is given by

(A8) $$\begin{array}{c} \begin{array}{c} \rho_{1}+\rho_{2}\frac{dp_{2}}{dp_{1}}=0. \end{array} \end{array}$$

Then,

(A9) $$\begin{array}{c} \begin{array}{cc} \frac{d}{dp_{1}}E_{M}\left[M^{2/\alpha }\right] & =\frac{2}{\alpha }\left(\rho_{1}p_{1}^{\frac{2}{\alpha }-1}+\rho_{2}p_{2}^{\frac{2}{\alpha }-1}\frac{dp_{2}}{dp_{1}}\right)\\ \; & \overset{\left(a\right)}{=}\frac{2}{\alpha }\rho_{1}\left(p_{1}^{\frac{2}{\alpha }-1}-p_{2}^{\frac{2}{\alpha }-1}\right)\overset{\left(b\right)}{<}0, \end{array} \end{array}$$

where (a) follows from (A8) and (b) follows from the assumption of $p_{1}>p_{2}$ and α>2. □

## Appendix F

**Proof of Theorem** **3.** For $\Omega =\Omega_{D}$, ${\bar{p}}_{E}={\left(\rho_{1}p_{1}^{2/\alpha }+\rho_{2}p_{2}^{2/\alpha }\right)}^{\alpha /2}$. Then, from (9) and (16), constraint (24b) can be expressed as $f_{q}\left({\bar{p}}_{E}\right)\geq \eta Q$. Here, $f_{q}\left(x\right)$ monotonically increases with *x* because it is obvious that $S_{E}$ increases with Tx power for a given ν. Therefore, as long as ${\bar{p}}_{E}\geq \theta$, (24b) is met. Because $p_{2}=\frac{\bar{p}-\rho_{1}p_{1}}{\rho_{2}}$ from constraint (24c), $f_{t}\left(p_{1}\right)$ is equivalent to ${\bar{p}}_{E}^{2/\alpha }$. According to Lemma 1, ${\bar{p}}_{E}^{2/\alpha }$ decreases as $p_{1}$ increases, indicating that $f_{t}\left(x\right)$ is a monotonic decreasing function with respect to *x*. Hence, ${\bar{p}}_{E}^{2/\alpha }$ is minimized when $p_{1}$ is maximized, i.e., when $p_{1}=\rho_{1}^{\alpha /2}\bar{p}$ and $p_{2}=0$ from constraint (24c). Hence, ${\bar{p}}_{E}$ is minimized at $\left(p_{1},p_{2}\right)=\left(\rho_{1}^{\alpha /2}\bar{p},0\right)$. As a result, if constraint (24b) is satisfied at the minimum value of ${\bar{p}}_{E}$, setting $\left(p_{1},p_{2}\right)=\left(\rho_{1}^{\alpha /2}\bar{p},0\right)$ leads to an objective function in (24a) that is unbounded above, thereby demonstrating that (26) holds. Conversely, if constraint (24b) is not met at the minimum value of ${\bar{p}}_{E}$, in order to both satisfy ${\bar{p}}_{E}\geq \theta$ and maximize (24a), $p_{1}$ should be increased until $f_{t}\left(p_{1}\right)=\theta^{2/\alpha }$, thereby ensuring (27) holds. □

## References

[1] Shen L.H., Feng K.T., Hanzo L. (2023). Five Facets of 6G: Research Challenges and Opportunities. ACM Compt. Surv..

[2] Wang C.X., You X., Gao X., Zhu X., Li Z., Zhang C., Wang H., Huang Y., Chen Y., Haas H. (2023). On the Road to 6G: Visions, Requirements, Key Technologies, and Testbeds. IEEE Commun. Surv. Tuts..

[3] Hayat O., Ngah R., Hashim S.Z.M., Dahri M.H., Malik R.F., Rahayu Y. (2019). Device Discovery in D2D Communication: A Survey. IEEE Access.

[4] Liu W., Nakauchi K., Shoji Y. (2018). A Neighbor-Based Probabilistic Broadcast Protocol for Data Dissemination in Mobile IoT Networks. IEEE Access.

[5] Alghamdi S.A. (2021). Cellular V2X With D2D Communications for Emergency Message Dissemination and QoS Assured Routing in 5G Environment. IEEE Access.

[6] Luo B., Yao Y., Sun Z. (2021). Performance Analysis Models of BLE Neighbor Discovery: A Survey. IEEE Internet Things J..

[7] Kindt P.H., Narayanaswamy S., Saur M., Chakraborty S. (2022). Optimizing BLE-Like Neighbor Discovery. IEEE Trans. Mob. Comput..

[8] Dhuli S., Atik F., Chhabra A., Singh P., Cenkeramaddi L.R. (2024). Performance Analysis of Gossip Algorithms for Large Scale Wireless Sensor Networks. IEEE Open J. Comput. Soc..

[9] Zhu Y., Liu M., Chen Y., Sun S., Li Z. (2024). SkyOrbs: A Fast 3-D Directional Neighbor Discovery Algorithm for UAV Networks. IEEE Trans. Mob. Comput..

[10] Bezerra L.C.D., Kouzayha N., Elsawy H., Bade A., Al-Naffouri T.Y. (2023). CSI-Based Proximity Estimation: Data-Driven and Model-Based Approaches. IEEE Open J. Commun. Soc..

[11] Qiu T., Chen B., Sangaiah A.K., Ma J., Huang R. (2018). A Survey of Mobile Social Networks: Applications, Social Characteristics, and Challenges. IEEE Syst. J..

[12] Ao W.C., Chen K.C. (2013). Error Control for Local Broadcasting in Heterogeneous Wireless Ad Hoc Networks. IEEE Trans. Commun..

[13] Kwon T., Choi J.W. (2014). Spatial Performance Analysis and Design Principles for Wireless Peer Discovery. IEEE Trans. Wirel. Commun..

[14] Kwon T., Ju H. (2020). Design Principles for Improving Spatial Secrecy Performance in Random Access based Wireless Mutual Broadcast Networks. IEEE Commun. Lett..

[15] Haenggi M. (2012). Stochastic Geometry for Wireless Networks.

[16] Lu X., Salehi M., Hossain E., Jiang H. (2021). Stochastic Geometry Analysis of Spatial-Temporal Performance in Wireless Networks: A Tutorial. IEEE Commun. Surv. Tuts..

[17] Baccelli F., Błaszczyszyn B., Mühlethaler P. (2006). An Aloha protocol for multihop mobile wireless networks. IEEE Trans. Inf. Theory.

[18] Baccelli F., Błaszczyszyn B., Mühlethaler P. (2009). Stochastic analysis of spatial and opportunistic aloha. IEEE J. Sel. Areas Commun..

[19] Hmamouche Y., Benjillali M., Saoudi S., Yanikomeroglu H., Renzo M.D. (2021). New Trends in Stochastic Geometry for Wireless Networks: A Tutorial and Survey. Proc. IEEE.

[20] Renzo M.D., Zappone A., Lam T.T., Debbah M. (2018). System-Level Modeling and Optimization of the Energy Efficiency in Cellular Networks–A Stochastic Geometry Framework. IEEE Trans. Wirel. Commun..

[21] Zheng T.X., Chen X., Wang C., Wong K.K., Yuan J. (2022). Physical Layer Security in Large-Scale Random Multiple Access Wireless Sensor Networks: A Stochastic Geometry Approach. IEEE Trans. Commun..

[22] Martin-Vega F.J., Soret B., Aguayo-Torres M.C., Kovacs I.Z., Gomez G. (2018). Geo-Location Based Access for Vehicular Communications: Analysis and Optimization via Stochastic Geometry. IEEE Trans. Veh. Technol..

[23] Wang R., Kishk M.A., Alouini M.S. (2024). Ultra Reliable Low Latency Routing in LEO Satellite Constellations: A Stochastic Geometry Approach. IEEE J. Sel. Areas Commun..

[24] Kwon T. (2014). Transmission Probability Design for Random Access Based Wireless Peer Discovery. IEEE Commun. Lett..

[25] Kwon T. (2015). Green Random Access for Wireless Peer Discovery. IEEE Commun. Lett..

[26] Kong H.B., Kang S., Lee I. (2017). Wireless Peer Discovery in Heterogeneous Half-/Full-Duplex Networks. IEEE Commun. Lett..

[27] Kang S., Kong H.B., Song C., Lee H., Lee I. (2017). Transmit Probability Designs for Wireless Peer Discovery with Energy Harvesting. IEEE Commun. Lett..

[28] Ashraf M., Kang S., Lee I. (2019). Harvested Energy Maximization in Wireless Peer Discovery Systems. IEEE Commun. Lett..

[29] Ashraf M., Lee K.G. (2019). Overall Energy Minimization in the Wireless Peer Discovery System with Multi-antenna Peers. IEEE Commun. Lett..

[30] Miyoshi N. (2022). Neveu’s Exchange Formula for Analysis of Wireless Networks With Hotspot Clusters. Front. Commun. Netw..

[31] Jiang J., Wang S., Han G., Wang H. (2023). Reinforcement-Learning-Based Adaptive Neighbor Discovery Algorithm for Directional Transmission-Enabled Internet of Underwater Things. IEEE Internet Things J..

[32] Shen Z., Gu C., Xiang X. (2023). Co-Circle: Energy-Efficient Collaborative Neighbor Discovery for IoT Applications. IEEE Internet Things J..

[33] Wei Z., Chen Q., Yang H., Wu H., Feng Z., Ning F. (2022). Neighbor Discovery for VANET With Gossip Mechanism and Multipacket Reception. IEEE Internet Things J..

[34] Wang J., Feng G., Qin S., Liu Y.-J., Zhou J., Peng Y., Wang Y., Zhang L. (2024). Intelligent Beam Configuration for Neighbor Discovery in Ad Hoc Networks with Directional Antennas. IEEE Trans. Veh. Technol..

[35] Seong H., Kim J., Shin W.Y., Lee H. (2023). FiFo: Fishbone Forwarding in Massive IoT Networks. IEEE Internet Things J..

[36] Guidoni D.L., Gottsfritz E.N., Meneguette R.I., Silva C.M., Filho G.P.R., Souza F.S.H. (2022). Toward an Efficient Data Dissemination Protocol for Vehicular Ad-Hoc Networks. IEEE Access.

[37] Chen L., Fan R., Zhang Y., Shi S., Bian K., Zhou P., Gerla M., Wang T., Li X. (2018). On heterogeneous duty cycles for neighbor discovery in wireless sensor networks. Ad Hoc Netw..

[38] Wei L., Chen Y., Zhang Y., Zhao L., Chen L. (2020). PSPL: A Generalized Model to Convert Existing Neighbor Discovery Algorithms to Highly Efficient Asymmetric Ones for Heterogeneous IoT Devices. IEEE Internet Things J..

[39] Kwon T., Ju H., LEE H. (2020). Performance Study for Random Access based Wireless Mutual Broadcast Networks with Ginibre Point Processes. IEEE Commun. Lett..

[40] Błaszczyszyn B., Karray M.K., Keeler H.P. Using Poisson Processes to Model Lattice Cellular Networks. Proceedings of the 2013 Proceedings IEEE INFOCOM.

